# Automated Determination of Nuclear Magnetic Resonance Chemical Shift Perturbations in Ligand Screening Experiments: The PICASSO Web Server

**DOI:** 10.1021/acs.jcim.1c00871

**Published:** 2021-11-29

**Authors:** Vincenzo Laveglia, Andrea Giachetti, Linda Cerofolini, Kevin Haubrich, Marco Fragai, Alessio Ciulli, Antonio Rosato

**Affiliations:** † Consorzio Interuniversitario di Risonanze Magnetiche di Metallo Proteine, Via Luigi Sacconi 6, 50019 Sesto Fiorentino, Italy; ‡ School of Life Sciences, Division of Biological Chemistry and Drug Discovery, The University of Dundee, James Black Centre, Dow Street, DD1 5EH, Dundee, United Kingdom; § Magnetic Resonance Center (CERM), University of Florence, Via Luigi Sacconi 6, 50019 Sesto Fiorentino, Italy; ∥ Department of Chemistry, University of Florence, Via della Lastruccia 3, 50019 Sesto Fiorentino, Italy

## Abstract

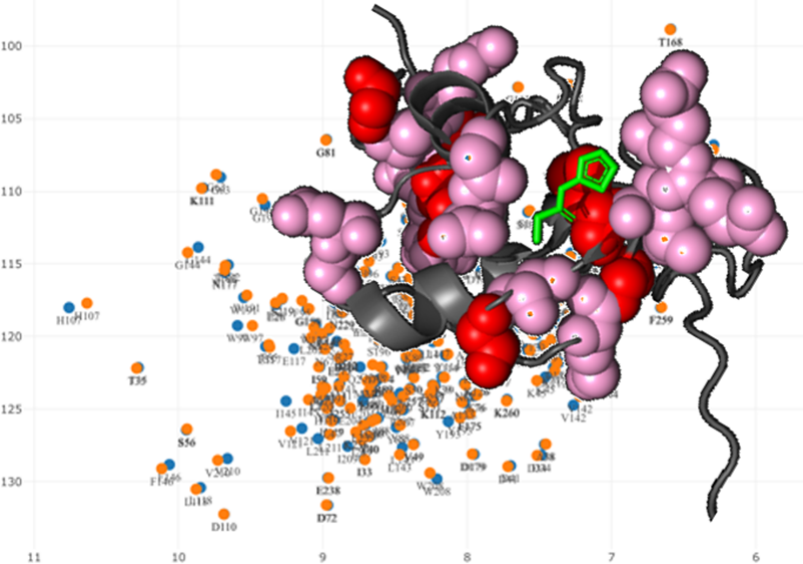

Nuclear magnetic resonance (NMR) is an effective, commonly used experimental approach to screen small organic molecules against a protein target. A very popular method consists of monitoring the changes of the NMR chemical shifts of the protein nuclei upon addition of the small molecule to the free protein. Multidimensional NMR experiments allow the interacting residues to be mapped along the protein sequence. A significant amount of human effort goes into manually tracking the chemical shift variations, especially when many signals exhibit chemical shift changes and when many ligands are tested. Some computational approaches to automate the procedure are available, but none of them as a web server. Furthermore, some methods require the adoption of a fairly specific experimental setup, such as recording a series of spectra at increasing small molecule:protein ratios. In this work, we developed a tool requesting a minimal amount of experimental data from the user, implemented it as an open-source program, and made it available as a web application. Our tool compares two spectra, one of the free protein and one of the small molecule:protein mixture, based on the corresponding peak lists. The performance of the tool in terms of correct identification of the protein-binding regions has been evaluated on different protein targets, using experimental data from interaction studies already available in the literature. For a total of 16 systems, our tool achieved between 79% and 100% correct assignments, properly identifying the protein regions involved in the interaction.

## Introduction

Interactions of proteins with other molecules define their cellular functions.^1^ These events are crucial for the proper functions of all processes in biological systems, and they are also relevant targets for the modulation of cellular process by drugs.^2,3^ An extensively used approach to develop new chemical probes to study biology as well as pharmaceuticals is screening small organic molecules (fragments) against a protein target to identify the interacting ligands and the residues forming the binding sites.^4^ Fragments are often weak binders with a binding specificity lower than expected from a lead compound.^5,6^ Thus, fragment-based drug discovery requires that the initial hits be further processed into lead compounds by chemical modification.

Nuclear magnetic resonance (NMR) spectroscopy is a very effective technique to get information about protein–ligand interactions at atomic resolution.^7−9^ In the protein-observed method, the spectrum of the target protein is acquired, and the ligand is titrated into the protein solution. This approach aims to provide information about the residues in the protein that are interacting with the ligand, either directly or through a modification of their environment induced by the binding event. The protein-observed method focuses on changes in the chemical shifts of the protein residues upon binding of the ligand. This is typically called the chemical shift perturbation (CSP) or chemical shift mapping (CSM) method. When the 3D structure of the protein is available, CSP data allow the identification of the interaction region on the protein surface and thus can drive docking calculations to derive a structural model of the protein:ligand adduct.^10−12^

Depending on the exchange rate between the free and bound species, NMR peaks change in position and/or shape during the titration of the target protein with the ligand in various manners.^13^ In the case of fast (with respect to the NMR time scale) exchange, the positions of the peaks vary according to the population-weighted average of the free and bound chemical shifts. Consequently, in the case of two-site exchange, each peak moves linearly from the position of the spectrum of the free protein to that of the adduct. In the case of slow exchange, separate peaks correspond to the free and bound states, with intensities proportional to the corresponding populations. In the case of intermediate exchange, line-broadening effects occur during the titration potentially leading to the disappearance of peaks from the spectrum. In all cases, tracking these chemical shift perturbations manually is tedious, especially when many peaks exhibit chemical shift changes and when many ligands should be tested. Automation of the tracking procedure via a variety of computational approaches has been described.^14−21^ These methods differ also in the type and quantity of experimental data required, from hand-picked peak lists of pairs of spectra to raw NMR data for all experiments in a titration series. None of these tools is available as a web server.

In this work, we developed a tool (PICASSO) requesting a minimal amount of experimental data from the user, implemented it as an open-source program, and made it available as a web application. PICASSO uses only two spectra, namely, those of the free protein and of the protein:ligand complex, represented by the corresponding peak lists. This avoids the need to upload the full spectrum but leaves the user with the burden to perform the peak picking procedure. Our approach minimizes the experimental information needed; when testing a series of ligands against a given protein target, it is sufficient to record one spectrum per protein:ligand adduct, plus the single reference of the free protein. The performance of PICASSO in terms of the correct identification of the protein-binding regions has been evaluated on different protein targets, using experimental data from published protein–ligand interaction studies.

## Methods

### Algorithms for Peak Assignments

The CSP (in ppm) of a given residue (*i*) is the weighted distance of its resonance peaks in the two ^1^H–^15^N HSQC spectra, defined as^22^
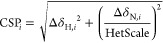
1where Δδ_H_ and Δδ_N_ are the chemical shift variations (in ppm) experienced by the ^1^H and ^15^N protein nuclei, respectively. HetScale is a scaling factor used to weight the heteronuclear shifts, due to the different origins of ^1^H vs ^15^N or ^13^C shifts.^22^ The HetScale factor has a default value of 5.0 in the PICASSO web server, which can be modified through the interface. Although some authors have suggested that a different weighting factor should be used for Gly residues,^23,24^ this is a relatively uncommon practice in the literature. Thus, for the sake of simplicity, we decided to use the same factor for all amino acids. Equation 1 can be straightforwardly used for ^1^H–^13^C HSQC spectra by replacing ^15^N with ^13^C chemical shift variations (i.e., Δδ_N_ with Δδ_C_), as well as other multidimensional spectra.^22^ In the following, we use the terms CSP and “peak distance” synonymously.

In all algorithms, we take as input two lists *P* = {*p*_1_, *p*_2_, ..., *p*_*n*_} and *P*′ = {*p*_1_′, *p*_2_′, ..., *p*_*m*_′} representing the positions of peaks in the 2D spectra of, respectively, the free protein and the protein in the presence of an equimolar amount or excess of ligand. The goal is to evaluate the CSPs caused by the ligand. As detailed below, this is all the input needed for the basic version of the two algorithms that we developed. In this case, the peaks in the input just need to be numbered sequentially and PICASSO will match the peaks in the two lists based on their distance. For the “smart” version of the algorithms, a further requirement is the assignments to the protein residues of all the peaks in one of the two lists (e.g., *P*). In this case, we can thus propagate the assignments from *P* to *P′*; unassigned peaks in *P* are neglected.

#### Sorting Distances (SD)

In this approach, we tried to emulate the human approach to the task, namely, first identify the closest pairs (*p*_*i*_, *p*_*j*_′), which most likely correspond to the same amino acid, then progressively assign all peaks based on proximity between the two sets, thus leaving the residues experiencing the largest CSPs as the last assignments. For this, we calculate the distances among all peaks in *P* to peaks in *P*′, resulting in a distance matrix , using eq 1. The smallest element in *D* corresponds to the first (*p*_*i*_, *p*_*j*_′) pair that is assigned. Then, row *i* and column *j* are removed from *D* and the procedure repeated until all peaks in the shortest list between *P* and *P′* have been matched.

#### Sorting Distances Smart (SDS)

In this improved version of SD, we take into account the distribution of CSPs along the sequence of the protein, based on the empirical observation that neighboring residues in the sequence tend to have CSPs of similar magnitude. We thus modified the algorithm in order to favor a situation where nearby residues have similar CSPs. For a peak *p*_*i*_ ∈ *P*, we identify the peak *p*_*j*_′ ∈ *P*′, such that the estimated CSP for the (*p*_*i*_, *p*_*j*_′) pair is similar to that of the neighborhood of residue *i*. The latter is defined through the *avg_csp*(*p*_*i*_, *k*) function, which calculates the average CSP around residue *i*, over a window range of size *k*. Consequently, here we want that *p*_*j*_′ is such that |CSP(*p*_*i*_, *p*′_*j*_) – *avg_csp*(*p*_*i*_, *k*)| is the smallest possible. The average CSPs are recalculated after each new assignment. The SDS assignment procedure is iterated until convergence, starting from the average CSP values of the previous iteration; for the first iteration, all CSPs are set to zero.

#### Resource Allocation (RA)

A completely different approach is that of looking at the CSP identification problem as a resource allocation (RA) problem. Given a set of tasks to be solved, and a set of available workers with a certain cost, RA approaches find task-worker allocation solutions that minimize the total cost. For the current application, a possible set up is considering the peaks in *P* as the tasks, the peaks in *P*′ as the workers, and the CSP as the cost of assigning *p*′_*j*_ to *p*_*i*_. In this scenario, solving the RA problem consists of finding the solution entailing the minimum CSPs. We implemented a well-known algorithm based on the documentation available at https://developers.google.com/optimization/assignment/overview.

#### Resource Allocation Smart (RAS)

In this approach, we added a refinement process to the RA algorithm. Given the assignments found by the RA algorithm and the corresponding set of CSPs, the goal is to refine the assignments in order to penalize configurations that feature high deviations from the local CSP average, as done in the SDS approach. On the basis of the RA results, for each *p*_*i*_ ∈ *P*, the average CSP of its neighbors is calculated with the aforementioned avg_csp(*p*_*i*_*,* k) function. Subsequently, the distances between *p*_*i*_ and all *p′*_*j*_ are updated as *d*_*ij*_ = *d*_*ij*_ /avg_csp(*p*_*i*_*, k*). The RA algorithm is then applied again using this updated distance matrix *D*, generating a new solution. This refinement procedure is repeated for a predefined number of times or until convergence is reached.

### Mapping of CSP Values onto X-ray Structures

To map the experimental and automated CSP values onto X-ray structures in a reproducible manner, we used a Python program to automatically build Pymol^25^ scripts. The protocol implemented was as follows:

(1) Compute the average and standard deviation (SD) of the CSP values for the whole protein.
(2) Remove residues having CSP values exceeding the whole protein average by at least four times the SD, as likely outliers.
(3) Define highly perturbed residues as those having CSP values exceeding the whole protein average by twice the SD.
(4) Define moderately perturbed residues as those having CSP values exceeding the whole protein average by at least one SD but not more than twice the SD.
(5) All heavy atoms of highly perturbed residues are displayed as red spheres of radius 1.0.
(6) All heavy atoms of moderately perturbed residues are displayed as pink spheres of radius 0.7.
(7) The protein backbone is displayed as a gray cartoon, whereas the ligand is shown as green sticks for small-molecule ligands or as a green cartoon for oligopeptide ligands.

Experimental and automatically generated CSP values were mapped independently. Note that the above protocol is only aimed at creating a reproducible graphical representation of the mapping. The data themselves are not affected, and the user is provided with the full unfiltered list of assignments and CSPs.

## Results

### Description of the Algorithms

We tested two different approaches to automatically transfer peak assignments from the free protein spectrum to the spectrum of the protein:ligand mixture. The input to our protocol is a pair of peak lists, usually from ^1^H–^15^N HSQC spectra. If an extensive assignment of all resonances is available, typically for the free protein, then PICASSO infers the assignments for the second peak list and hence derives the CSPs, using the SDS or RAS algorithms. In the absence of such assignments, PICASSO uses the SD and RA algorithms to identity peak displacements based on the minimization of the overall CSP. Consequently, peaks from the first list are matched to peaks in the second list but not to the protein sequence.

In the SD method, we tried to replicate the human approach to the task, namely, assigning peaks with no or very little shift first and then trying to resolve by proximity the assignment of more shifted peaks. This approach tends to minimize the sum of all CSPs, which is known to have some shortcomings, for example, when the signal(s) from one (or more) residue(s) is missing in either spectrum or when two nearby peaks experience very different shifts upon addition of the ligand.^14^ We therefore introduced a correction, based on the empirical observation that in well-behaved systems residues having significant CSPs tend to cluster along the sequence. We thus replaced the proximity metrics with a function that depends on the deviation of the CSP of each residue from the average CSP of its sequence neighbors, calculated over a window of seven residues. This refined approach (SDS) penalizes isolated residues featuring high CSP values within sequence stretches with small CSPs and vice versa. The SDS method can be applied only if the residue assignments of the free protein are available, in order to be able to identify the sequence neighbors of each residue.

For the design of our second approach (RA), we formulated the algorithm as a resource allocation problem, in which a number of agents (the peaks from the first spectrum) are assigned to a number of tasks (the peaks from the second spectrum), incurring a cost that depends on the agent–task assignment (the corresponding CSP). The goal is to perform as many tasks as possible by assigning at most one agent to each task and at most one task to each agent, at a minimum total cost. To go beyond the simple approach of minimizing the sum of all CSPs, we also implemented a “smart” version of algorithm RAS, incorporating the concept of local similarity along the sequence described above.

### Validation against Experimental Data

We ran our algorithms on 16 experimental systems, from five different proteins (Table 1). For these systems, chemical shift assignments were available for both the free protein and the protein in the adduct, obtained through multidimensional NMR methods. Our test set included both chemically complex inhibitors and smaller molecules that would be used in fragment screening experiments. We measured the accuracy of the algorithms as the percentages of the peaks predicted for the spectrum of the protein:ligand adduct that have been correctly assigned. By taking the best solution of any algorithm, this value ranged from 79% to 100%. The RAS algorithm had the best results (Supporting Information), with an average performance of 94%.

**Table 1:** Experimental Systems Used for Validation and Corresponding Performance of the Algorithm^a^

Protein	Ligand	ref	SD (%)	SDS (%)	RA (%)	RAS (%)	Best (%)
MMP-12	1. N-isobutyl-N-[4methoxyphenylsulfonyl] glycyl hydroxamic acid	(26)	81	**82**	79	81	82
MMP-12	2. N-[(2S)-3-[(S)-(4-bromophenyl)(hydroxy)phosphoryl]-2-{[3-(3′-chlorobiphenyl-4-yl)-1,2-oxazol-5-yl]methyl}propanoyl]-L-alpha-glutamyl-L-alpha-glutamine	(27)	72	73	73	**79**	79
Carbonic anhydrase II	3. Furosemide	(28)	95	94	95	**97**	97
Carbonic anhydrase II	4. Oxalate	(29)	93	91	95	**96**	96
Carbonic anhydrase II	5. p-Toluenesulfonamide	Table S2	92	92	98	**99**	99
Carbonic anhydrase II	6. p-Toluenesulfonic acid	Table S3	**100**	**100**	**100**	**100**	100
Carbonic anhydrase II	7. Thiocyanate	Table S4	80	81	90	**92**	92
Carbonic anhydrase II	8. Sulpiride	(30)	89	89	98	**100**	100
BAZ2A	9. ARTKQTARKS decapeptide	(31)	79	72	70	**83**	83
BAZ2A	10. ARTKQ pentapeptide	(31)	85	89	79	**96**	96
BAZ2B	10	(31)	**91**	87	79	89	91
Ube2T	11. 6-Chloro-2,3,4,9-tetrahydro-1H-carbazole-1-carboxamide (EX-527)	(32)	99	99	99	**100**	100
Ube2T	12. 1,3-Benzothiazol-2-ylmethylamine hydrochloride	(32)	96	**97**	96	**97**	97
Ube2T	13. 3,4-Dihydro-3-methyl-2(1H)-quinazolinone	(32)	**98**	**98**	**98**	**98**	98
Ube2T	14. 2-Amino-5-phenyl-3-furonitrile	(32)	95	**96**	**96**	**96**	96
Ube2T	15. 5-(2-Pyridyl)thiophene-2-carboxamide	(32)	94	**98**	94	**98**	98
Average			90	90	90	**94**	94
Median			93	92	95	**97**	97

a HetScale (eq 1) was 5.0 for all calculations. Bold numbers indicate the best performing algorithm.

**Figure 1:**
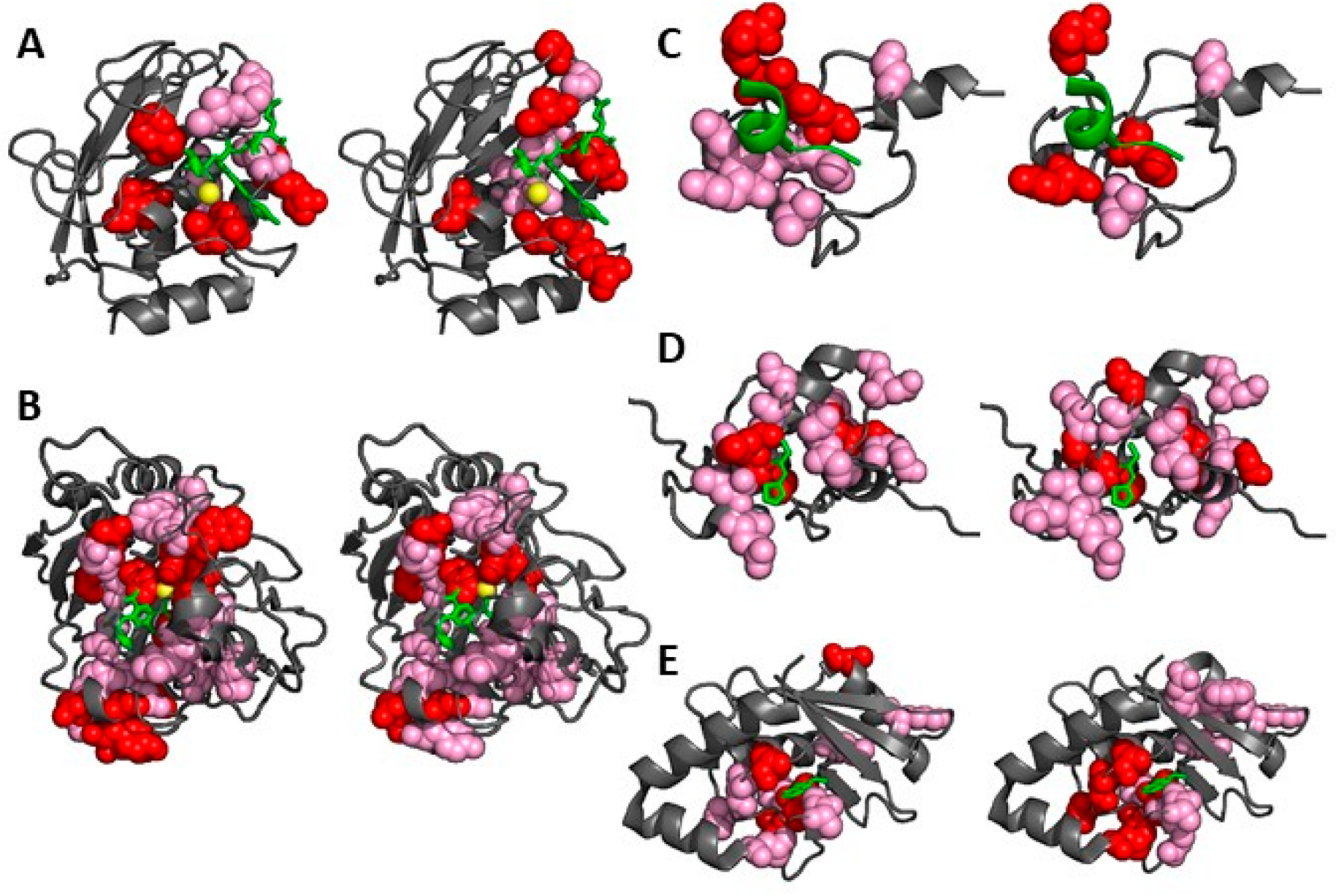
Comparison of the mapping of experimental (left) and automatically derived CSPs (right) for (A) the MMP-12:**2** adduct mapped onto the corresponding X-ray structure (PDB entry 4GQL), (B) the carbonic anhydrase II:**3** adduct mapped onto the corresponding X-ray structure (PDB entry 1Z9Y), (C) the BAZ2A:**9** adduct mapped onto the corresponding X-ray structure (PDB entry 5T8R), (D) the BAZ2B:**10** adduct mapped onto the X-ray structure of a related adduct (PDB entry 6FHQ), and (E) the Ube2T:**12** adduct mapped onto the corresponding X-ray structure (PDB entry 5NGZ). All heavy atoms of residues experiencing a CSP exceeding the protein average by at least one SD are shown as spheres; residues with a CSP exceeding the protein average by two SD or more are colored in red, whereas residues with a CSP exceeding the protein average by less than two SD are colored in pink. The ligand is shown in green; the protein backbone is shown as a gray cartoon. The catalytic zinc ions in (A) and (B) are shown as yellow spheres. The accuracies of the automated assignments were, respectively, 79.0%, 96.7%, 83.0%, 88.9%, and 97.3%.

However, Table 1 does not provide the full picture about the usefulness of the predictions. For example, swapping two peaks that shift modestly upon addition of the ligand will not affect the subsequent analysis of the experiments. Instead, it is important to have a good mapping of the residues that define whether a detectable interaction occurs and allow the 3D configuration of the protein:ligand adduct to be modeled. Traditionally, one selects an arbitrary threshold of CSP to identify residues that are affected more than the rest of the protein, assuming that they are in close spatial proximity to the ligand. Then, if a 3D structure of the receptor is available, their position is mapped on the protein surface, providing a visual representation of where the interaction happens. Figure 1 compares the mapping of the experimental and automated CSP values of one adduct for each protein on the X-ray structures of the same systems, except for BAZ2B where we used a related structure. The experimental and automated mappings agree from well to very well for all systems. The mappings were generated automatically (see Methods) to avoid any arbitrary choice. Interestingly, as the CSPs for some of these systems are quite small, changing only one or two assignments that involve the residues with the larger CSPs can change the average and standard deviation parameters as to involve an appreciably different number of residues in the mapping; an example of this is the E2 conjugating enzyme Ube2T in Figure 1E.

### Tool Availability

We provide access to the tool (PICASSO) via a web server at https://picasso.cerm.unifi.it/, maintained at the University of Florence. The web server is based on a combination of React (https://reactjs.org) for the frontend and Spring Boot (https://spring.io/projects/spring-boot) for the backend technologies. The input should be provided as csv (comma-separated values) files, with the organization of the columns shown in Table S1 and on the web server itself.

**Figure 2:**
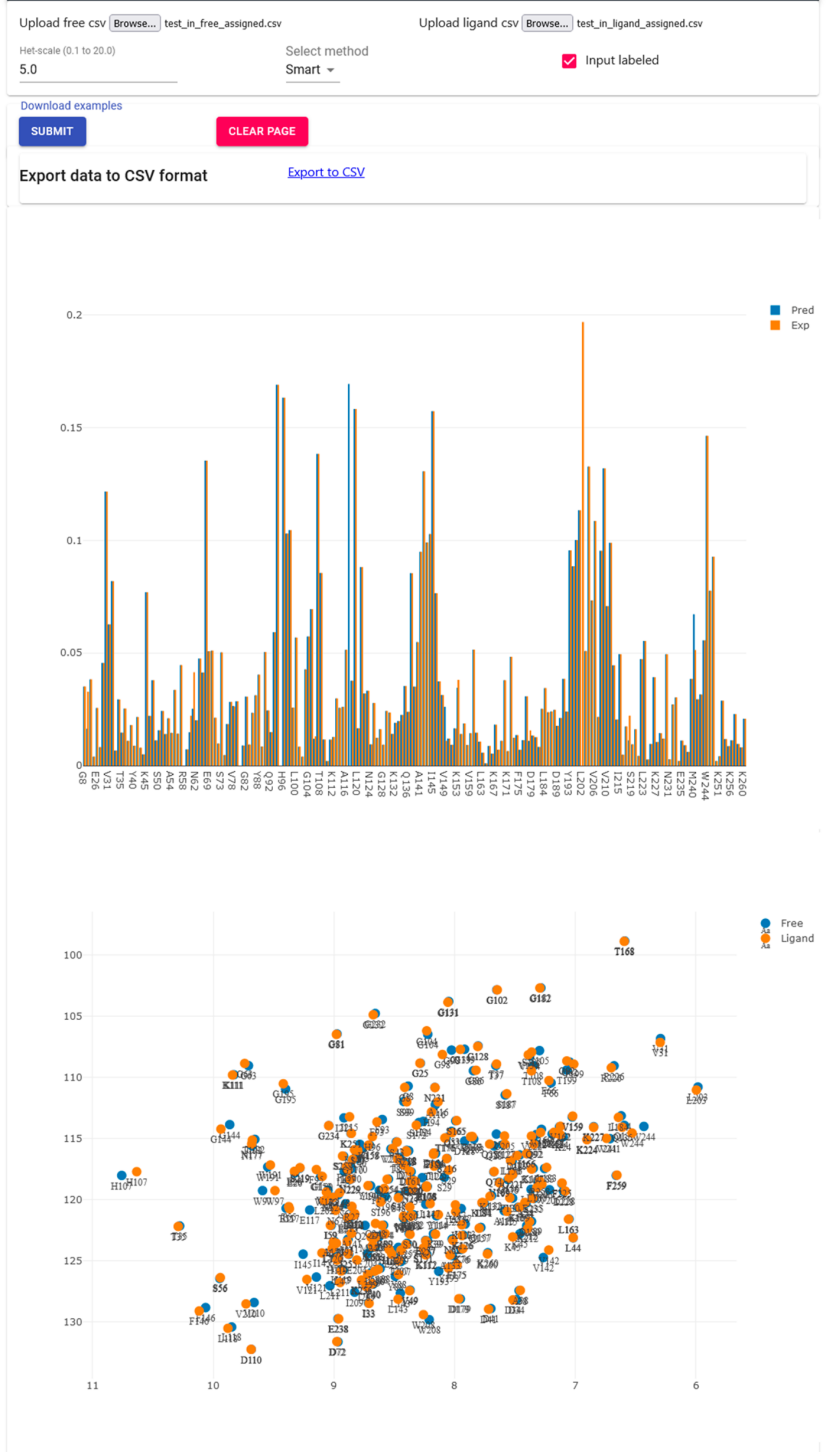
Output page of the PICASSO web server.

The web server provides the user with the predictions computed by either the RAS algorithm (method: Smart) or the RA algorithm (method: Proximity). The Smart method requires residue assignments to be included in the peak list of the free protein. The Proximity method permits the use of PICASSO in the absence of assignments. Regardless of the method chosen, the input value of the HetScale parameter (set to 5.0 as the default value) is used according to eq 1. The results page displays a histogram of the predicted CSPs on a per-residue basis together with a downloadable table of the predicted assignments/peak matches and the corresponding CSP values (Figure 2). If both input lists include the peak assignments, then the user can additionally request a comparison of the predicted vs previously determined assignments by checking the “Input labeled” box.

## Discussion

In this work, we developed a tool, called PICASSO, that automatically transfers protein assignments from a 2D heteronuclear NMR spectrum, for example, ^1^H–^15^N HSQC, of a free protein sample (reference spectrum) to the corresponding spectrum acquired on a protein:ligand mixture (target spectrum). This is done by selecting the Smart method of the web server (Figure 2). PICASSO can be used also in the absence of any assignments (selecting the Proximity method) in order to identify binders in a screening experiment, depending on whether a group of peaks has moved beyond a given threshold. The implemented procedure assumes that the number of peaks in the two spectra will be similar. Thus, it is applicable to systems with fast chemical exchange between the free protein and the protein–ligand adduct or to systems in the slow exchange regime under experimental conditions where the protein is saturated with the ligand, namely, a suitable molar excess of the ligand. For systems in the fast exchange, the protein will always display a single set of signals, whereas in the slow exchange, there will be two sets of signals, with intensity proportional to the molar fraction of the free and ligand-bound protein. Using an excess of ligand in the mixture pushes the molar fraction of the adduct close to 1, making the free protein undetectable. An excess of ligand is beneficial also for systems in the fast exchange, inducing larger CSPs.^22^ Using only two spectra is sufficient for PICASSO to perform reliably; however, more data are required to distinguish between slow and fast exchanges.

Table 1 shows that in our test systems the protein:ligand ratios used ranged from 1:1 for nanomolar enzyme inhibitors where the formation of the adduct is practically quantitative and the exchange regime is slow, up to 1:1250 for fragments in the fast exchange. PICASSO can be applied also to cases where both the reference and target spectra contain ligands, typically with different affinity to the protein, as done for the two MMP12 samples (Figure 1), where the reference spectrum contains acetohydroxamic acid to prevent autoproteolysis.

The aim of our work was to provide a simple tool enabling a ready mapping of the protein–ligand interaction, viewed from the protein side through 2D HSQC spectra. This approach is relatively low throughput, but it has the advantage of providing structural information on the protein–ligand adduct. To highlight this aspect, we mapped the predicted CSP values onto the protein surfaces of selections from our test cases, preferentially choosing those systems for which crystallographic 3D structures are available with the same ligand (Figure 1). On the basis of the comparison with the manual assignments, it appears that PICASSO correctly identified all binding sites although with some variation in the specific residues singled out. The accuracy for the examples shown in the figures ranges from 79% to 97% (Table 1). The distinction among highly and moderately perturbed residues in Figure 1 (red and pink color, respectively) is practically not relevant for many docking algorithms, which would simply require a list of residues whose peaks have moved beyond an arbitrary threshold value.

Our application exploits an algorithmic approach that is simpler than other tools previously described in the literature. Our aim in developing the PICASSO tool was mainly to provide a user-friendly tool that did not require any local installation nor explicitly designed experimental schemes. Hence, PICASSO uses a pair of simple HSQC experiments and is available as a web server (https://picasso.cerm.unifi.it/). PICASSO is less versatile than tools such as CSP Analyzer,^21^ which leverages machine learning to analyze extensive series of peak lists against a reference peak list to automatically identify binding events based only on the changes in the appearance of each list (i.e., without having to perform assignments). Another relevant approach was the Automatic PEak Tracking (APET) algorithm,^14^ which allowed mapping reference and ligand-containing spectra with sophisticated mathematical tools. This is only available as a module in the FELIX suite, distributed by Felix NMR, Inc. A related algorithm is PROPET, to analyze titration series.^14^ Similarly, the NvMap tool^16^ is a module of the NMRViewJ package^33^ using a greedy algorithm to sequentially match peak couples from the reference and target peak lists, generated using NMRViewJ, based on their distance. There are also tools available as stand-alone programs, such as PeakWalker^18^ or GAPT.^17^ PeakWalker is a Java program available from the authors that allows a peak to be followed through a series of spectra. GAPT is a Visual Basic program that also enables the automated tracking of peak trajectories along a titration series, through graph search methods.^17^ A more recent tool is Trace in Track (TinT),^19^ which also deals with a series of NMR spectra where a stepwise perturbation is applied (e.g., a ligand titration). Finally, a quite recent platform that incorporates, among many other tools, the capability of determining CSPs and mapping them onto 3D protein structures is the POKY suite,^34^ the successor of NMRFAM-SPARKY.^35^ These are very articulated program suites, encompassing tasks from the interactive analysis of spectra to resonance assignments and automated 3D structure determination, written over several years in different computer languages.^34,35^ In summary, the discussion above highlights how the currently available tools are not of immediate availability to users; some require the local installation of a program, upon request to the authors, whereas others are embedded in complex, sometimes commercial, suites. The source codes are typically not readily available. Furthermore, some tools focus exclusively on the reconstruction of peak trajectories along a titration series. This is a different problem than the one tackled by PICASSO, i.e., transferring assignments from the reference to the target spectrum. Besides the additional amount of experimental information needed, the analysis of peak trajectories is not suitable to address systems in the slow exchange regime.^18^

PICASSO is available open access and without registration at https://picasso.cerm.unifi.it/. There are two main lines for future development: (i) integration with automated procedures for HSQC spectra processing and peak picking, for example, using NMRpipe^36^ as the main software package and Nextflow^37^ as a lightweight workflow management system and (ii) incorporating 3D structural information. Our tool is entirely written in Python, which permits easy integration with other packages in the same language, such as nmrglue,^38^ a Python-based module for working with NMR data. Currently, users must produce the input peak lists for PICASSO independently; to facilitate this task, our tool requires input in the csv format, which can be produced by most software used to handle NMR data. Thus, there is no need for a specific software upstream. Peak picking can be performed through the software interface of the spectrometer, the NMR processing package adopted in the lab, or a separate peak picking tool.^39,40^

## Data and Software Availability

The source code of PICASSO is freely available at https://github.com/cerm-cirmmp/picasso. Test data were taken from the cited publications or are available as Supporting Information.

## Supporting Information Available

Figures S1–S16: Comparison of experimental vs predicted (with the *RAS* algorithm) CSPs for all test systems. Table S1: Format of input csv data to the Picasso web server. Table S2: Experimental 1H and 15N chemical shift values of carbonic anhydrase II in the presence of p-toluenesulfonamide in a 1:1 molar ratio with respect to the protein. Table S3: Experimental 1H and 15N chemical shift values of carbonic anhydrase II in the presence of p-toluenesulfonic acid in a 100:1 molar ratio with respect to the protein. Table S4: Experimental 1H and 15N chemical shift values of carbonic anhydrase II in the presence of thiocyanate in a 1250:1 molar ratio with respect to the protein. (PDF)
