# Supplementary material for: Automated Determination of Nuclear Magnetic Resonance Chemical Shift Perturbations in Ligand Screening Experiments: The PICASSO Web Server
Source: J Chem Inf Model. 2021 Nov 29;61(12):5726–33. doi: 10.1021/acs.jcim.1c00871 (PMC8715503; doi:10.1021/acs.jcim.1c00871)
Supplement: Supplementary file 1 — ci1c00871_si_001.pdf [file ci1c00871_si_001.pdf]

Automated determination of NMR chemical shift perturbations in ligand screening experiments: the PICASSO webserver

Vincenzo Laveglia[1§], Andrea Giachetti[1§], Linda Cerofolini[1], Kevin Haubrich[2], Marco Fragai[1,3,4], Alessio Ciulli[2], Antonio Rosato[1,3,4,*]

§ Contributed equally

1 Consorzio Interuniversitario di Risonanze Magnetiche di Metallo Proteine, Via Luigi Sacconi 6, 50019 Sesto Fiorentino, Italy.
2 School of Life Sciences, Division of Biological Chemistry and Drug Discovery, The University of Dundee, James Black Centre, Dow Street, DD1 5EH, Dundee, United Kingdom
3 Magnetic Resonance Center (CERM), University of Florence, Via Luigi Sacconi 6, 50019 Sesto Fiorentino, Italy.
4 Department of Chemistry, University of Florence, Via della Lastruccia 3, 50019 Sesto Fiorentino, Italy.
Corresponding author: Dr. Antonio Rosato, rosato@cerm.unifi.it

Supporting information

Supporting Figures S1-S16: Comparison of experimental vs predicted (with the *RAS* algorithm) CSPs for all test systems

Supporting Table S1. Format of input csv data to the Picasso web server.

Supporting Table S2. Experimental ^{1}H and ^{15}N chemical shift values of carbonic anhydrase II in the presence of p-toluenesulfonamide in a 1:1 molar ratio with respect to the protein.

Supporting Table S3. Experimental ^{1}H and ^{15}N chemical shift values of carbonic anhydrase II in the presence of p-toluenesulfonic acid in a 100:1 molar ratio with respect to the protein.

Supporting Table S4. Experimental ^{1}H and ^{15}N chemical shift values of carbonic anhydrase II in the presence of thiocyanate in a 1250:1 molar ratio with respect to the protein.

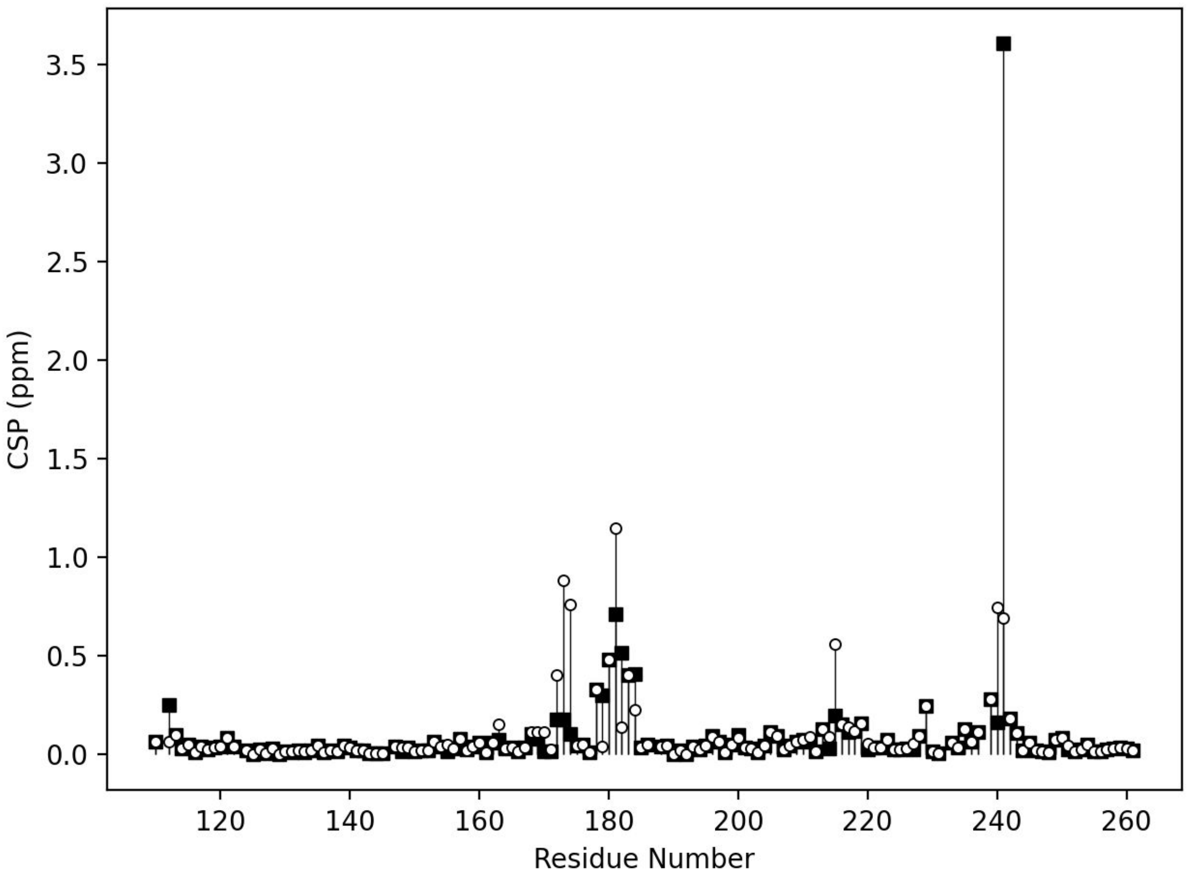

Supporting Figure S1 Comparison of experimental (black squares) and predicted (open circles) CSPs upon addition of ligand 1 to MMP-12.

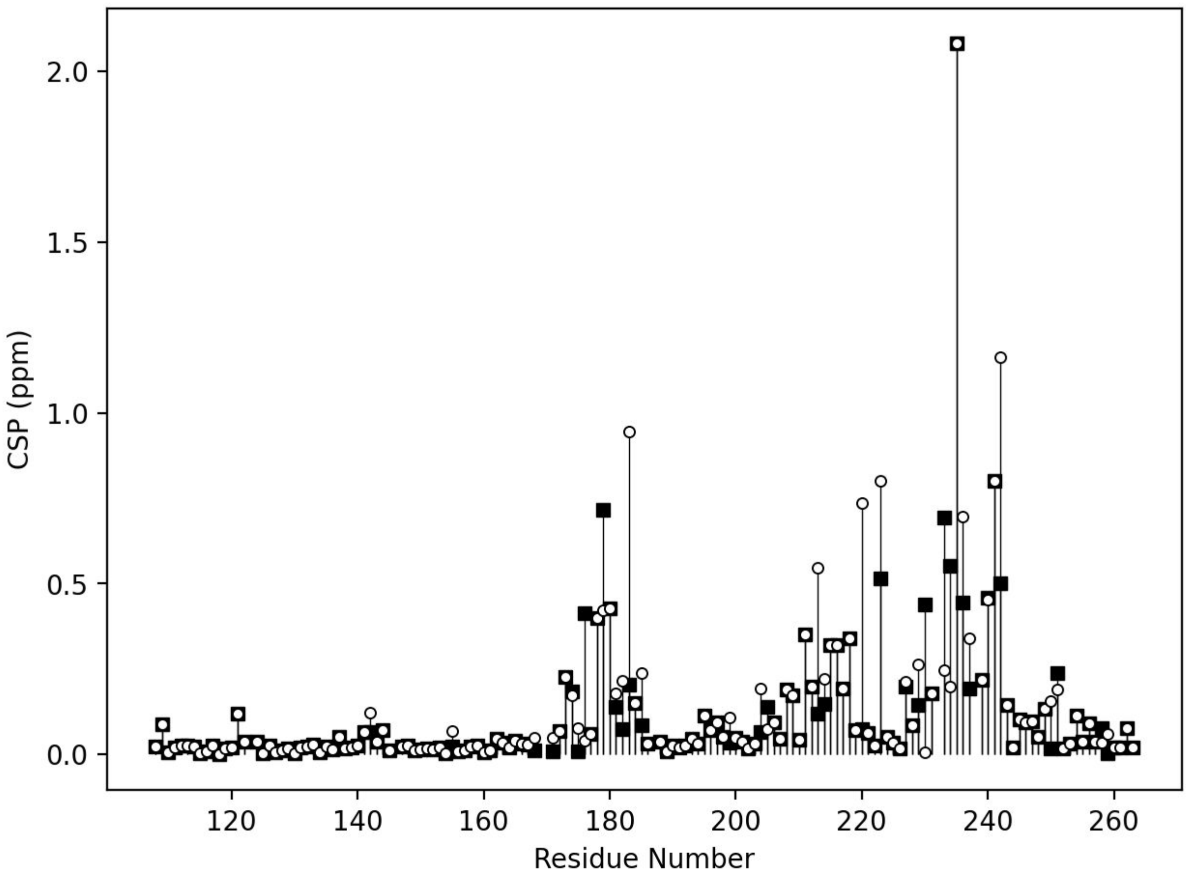

Supporting Figure S2 Comparison of experimental (black squares) and predicted (open circles) CSPs upon addition of ligand 2 to MMP-12.

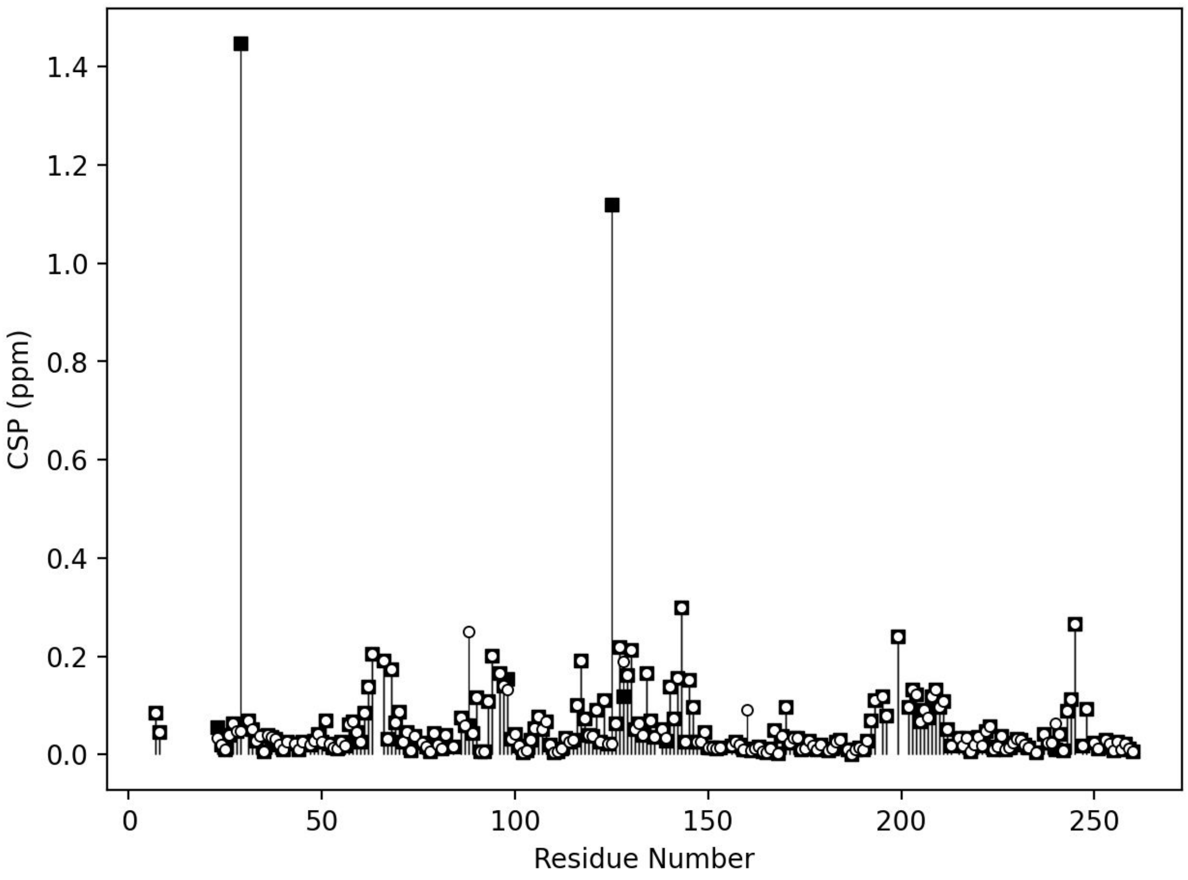

Supporting Figure S3 Comparison of experimental (black squares) and predicted (open circles) CSPs upon addition of ligand 3 to human carbonic anhydrase II

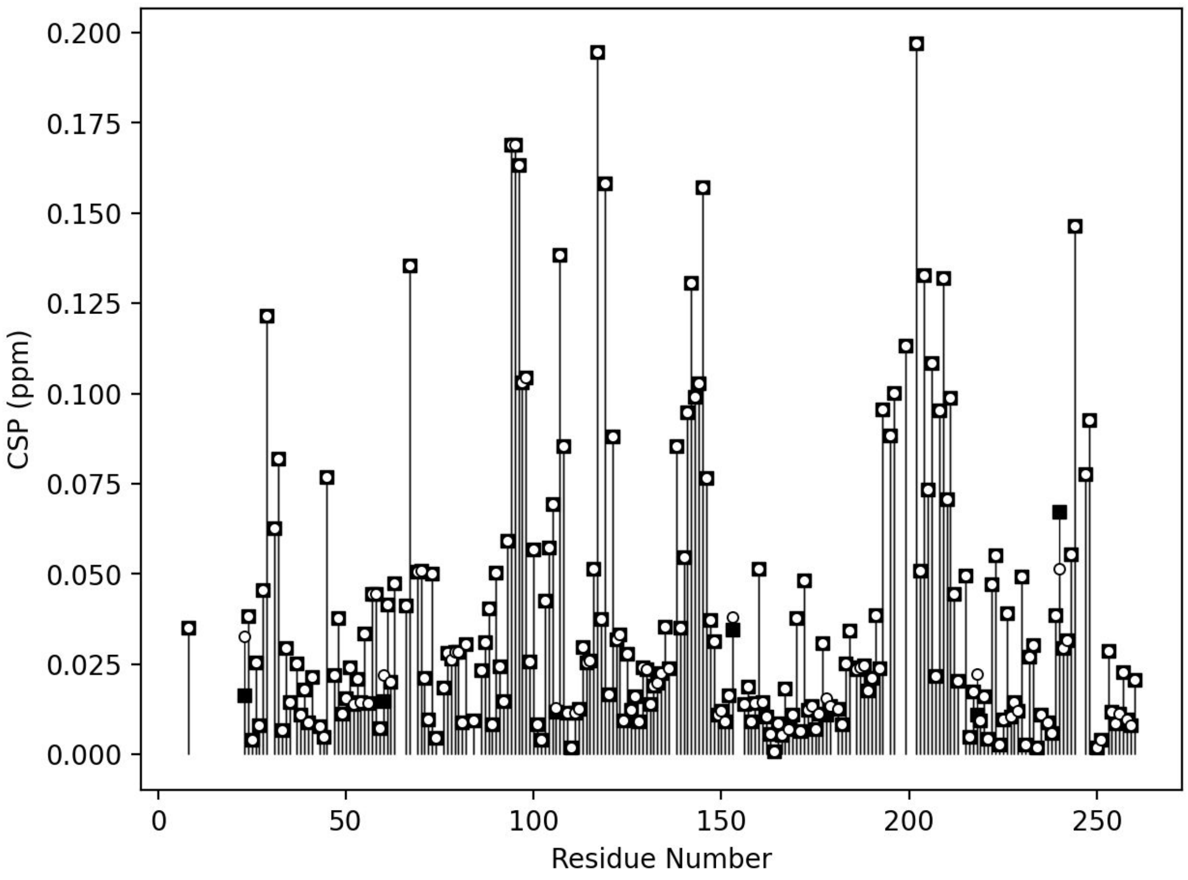

Supporting Figure S4 Comparison of experimental (black squares) and predicted (open circles) CSPs upon addition of ligand 4 to human carbonic anhydrase II

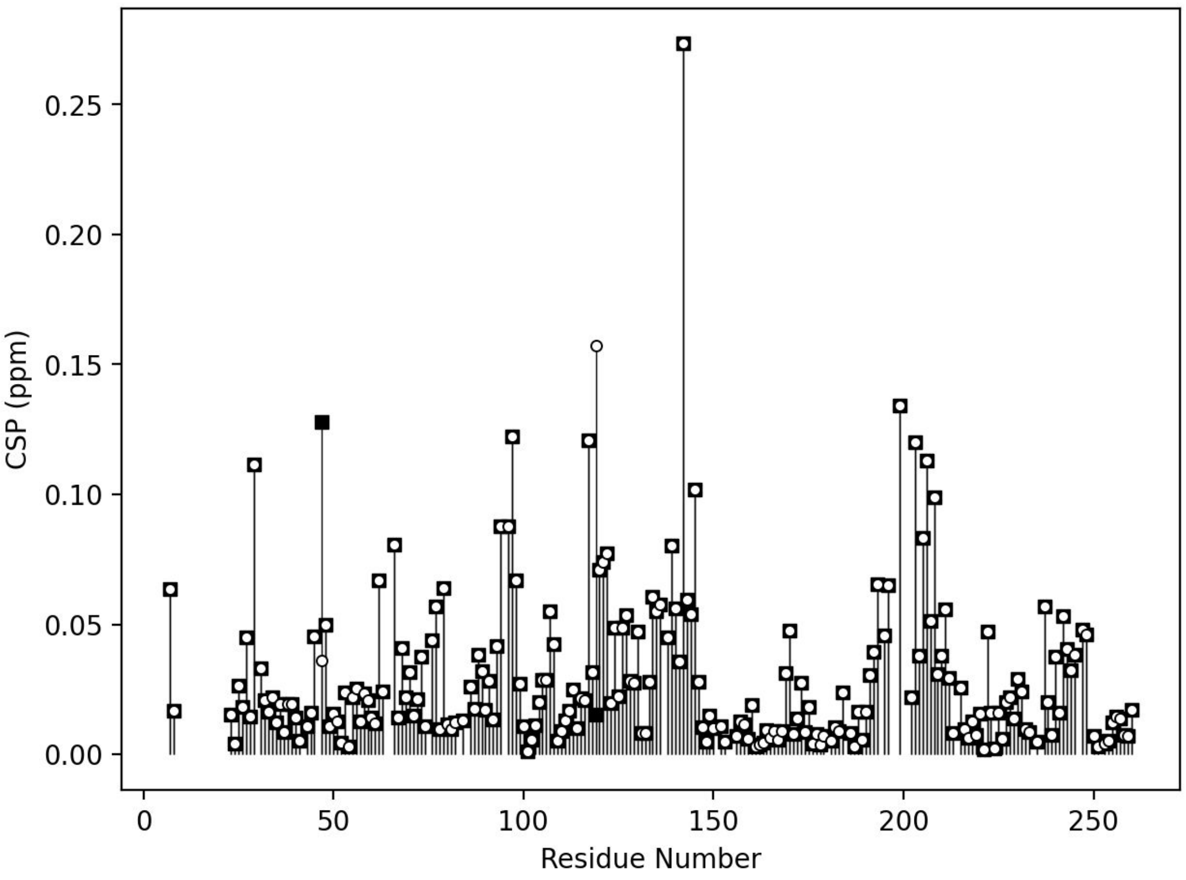

Supporting Figure S5 Comparison of experimental (black squares) and predicted (open circles) CSPs upon addition of ligand 5 to human carbonic anhydrase II

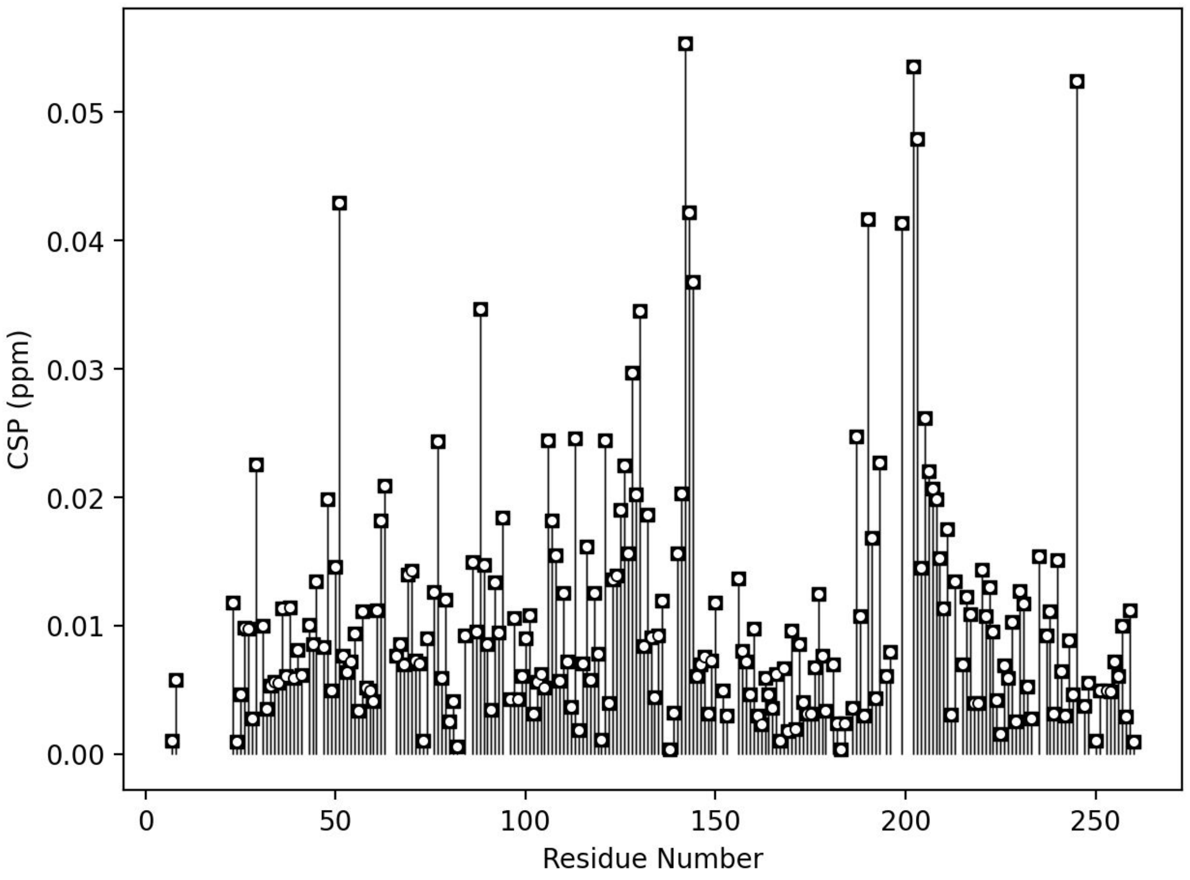

Supporting Figure S6 Comparison of experimental (black squares) and predicted (open circles) CSPs upon addition of ligand 6 to human carbonic anhydrase II

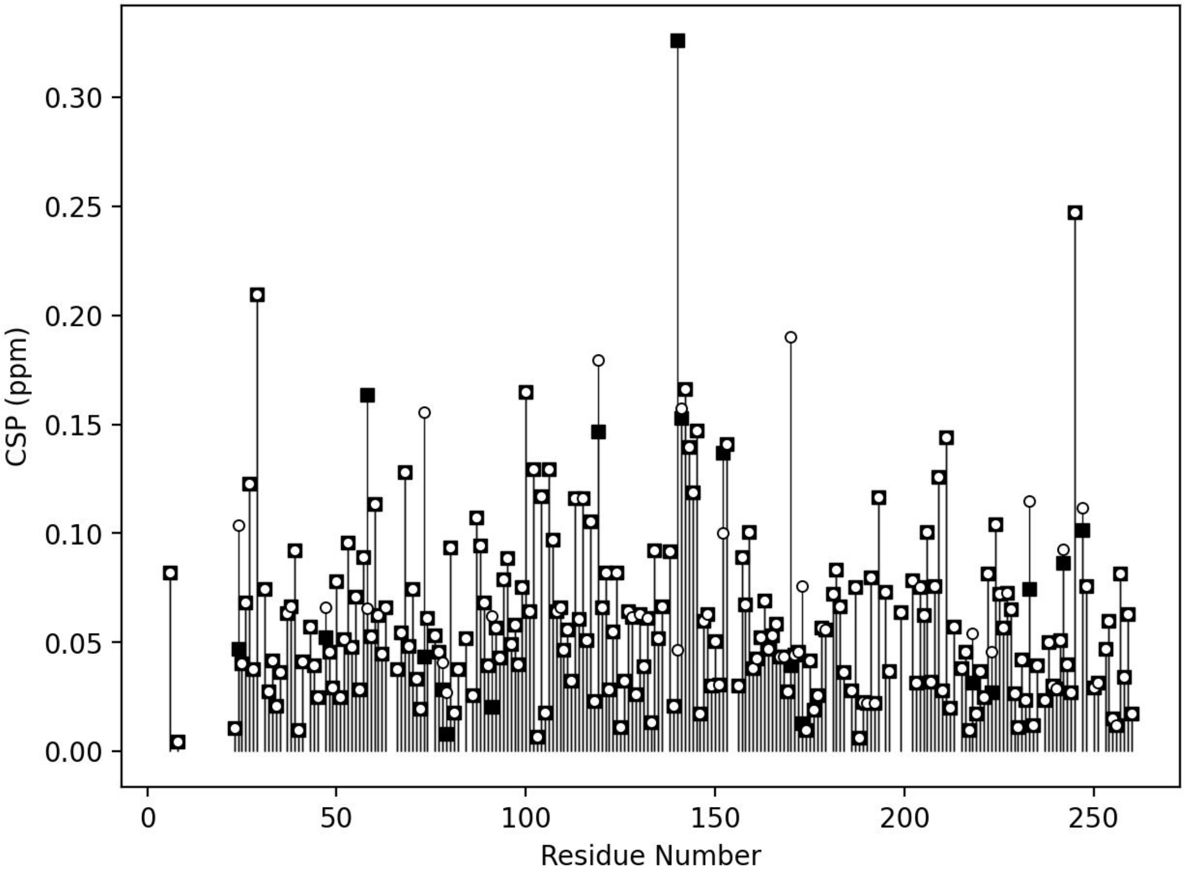

Supporting Figure S7 Comparison of experimental (black squares) and predicted (open circles) CSPs upon addition of ligand 7 to human carbonic anhydrase II

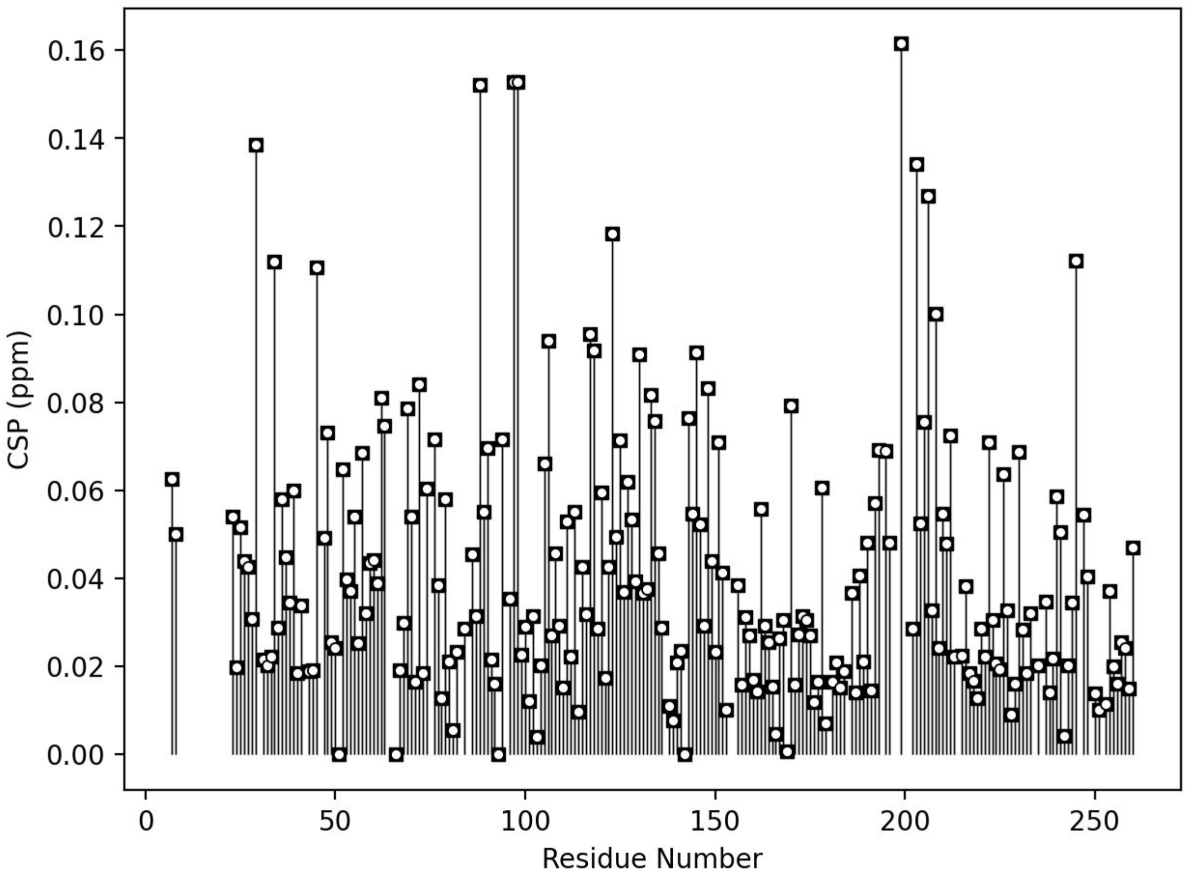

Supporting Figure S8 Comparison of experimental (black squares) and predicted (open circles) CSPs upon addition of ligand 8 to human carbonic anhydrase II

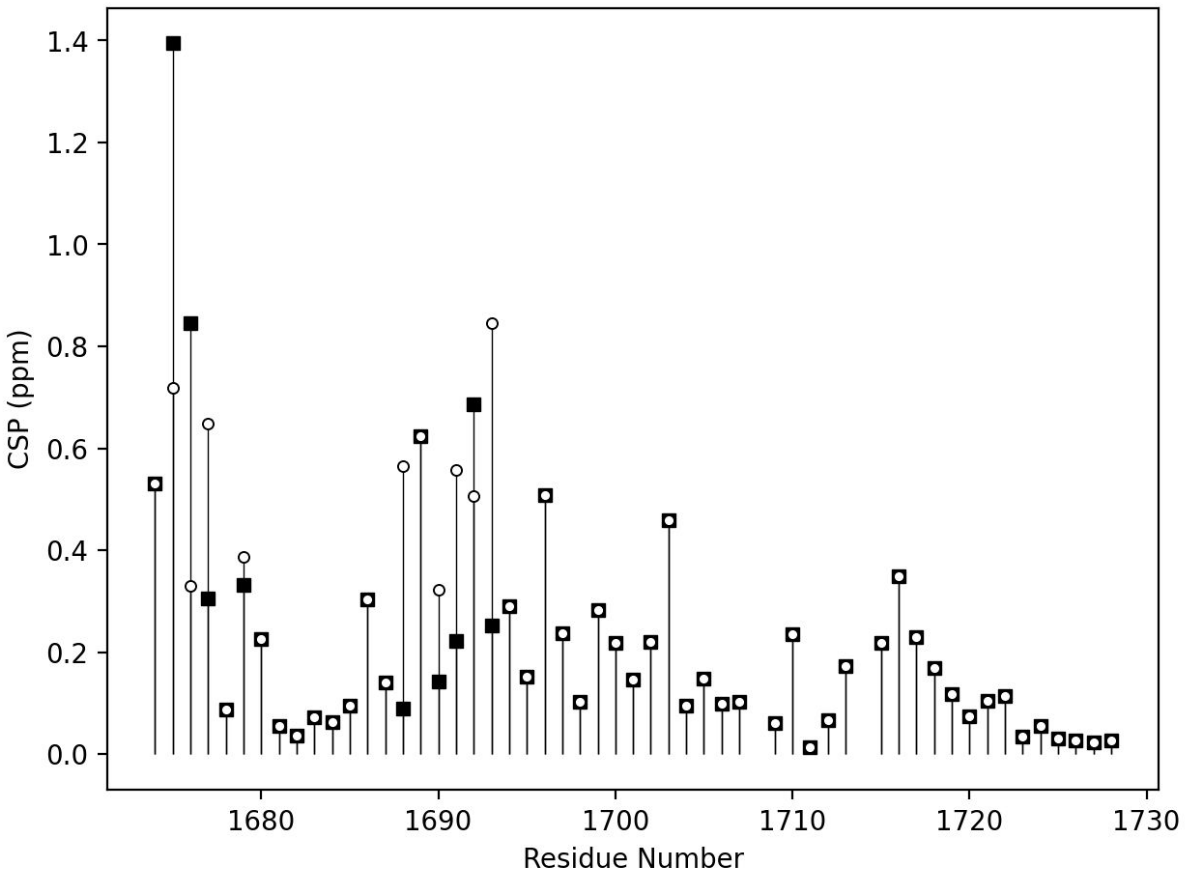

Supporting Figure S9 Comparison of experimental (black squares) and predicted (open circles) CSPs upon addition of ligand 9 to BAZ2A

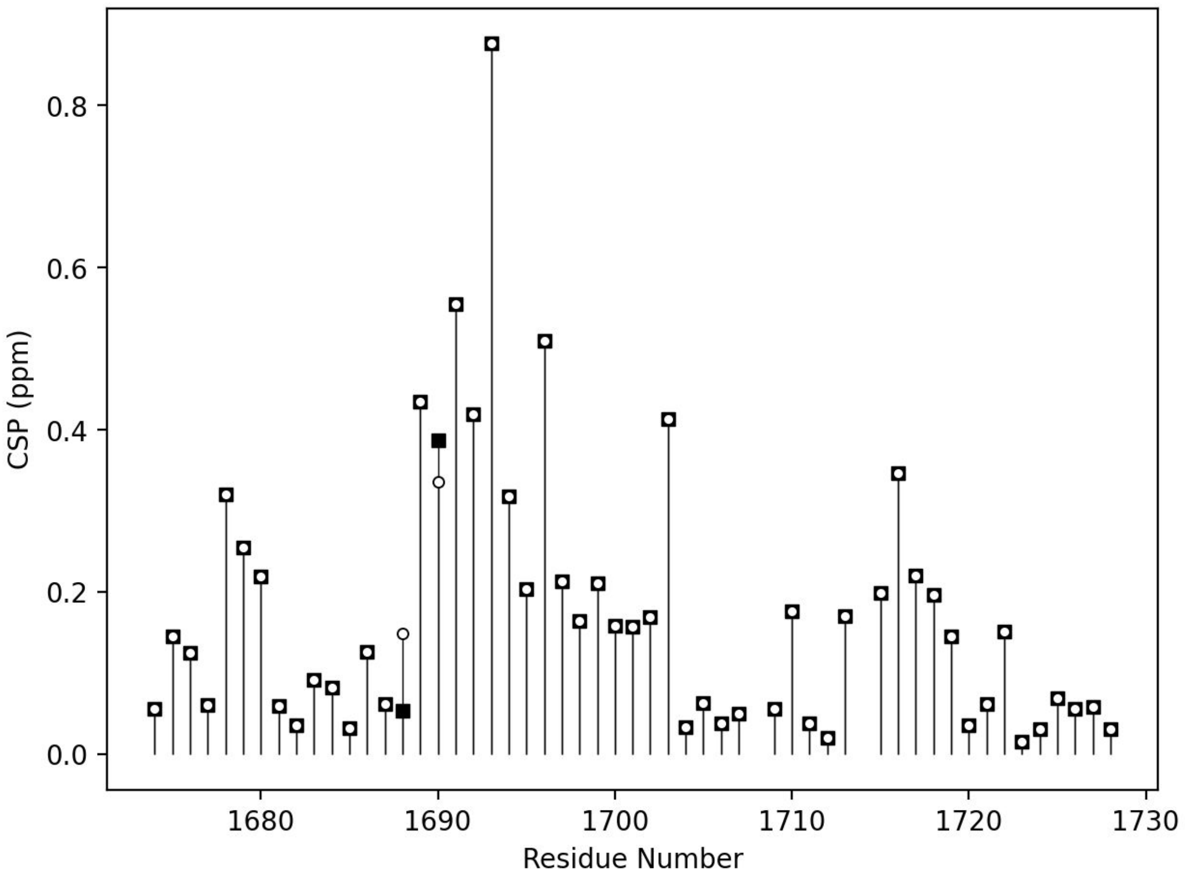

Supporting Figure S10 Comparison of experimental (black squares) and predicted (open circles) CSPs upon addition of ligand 10 to BAZ2A

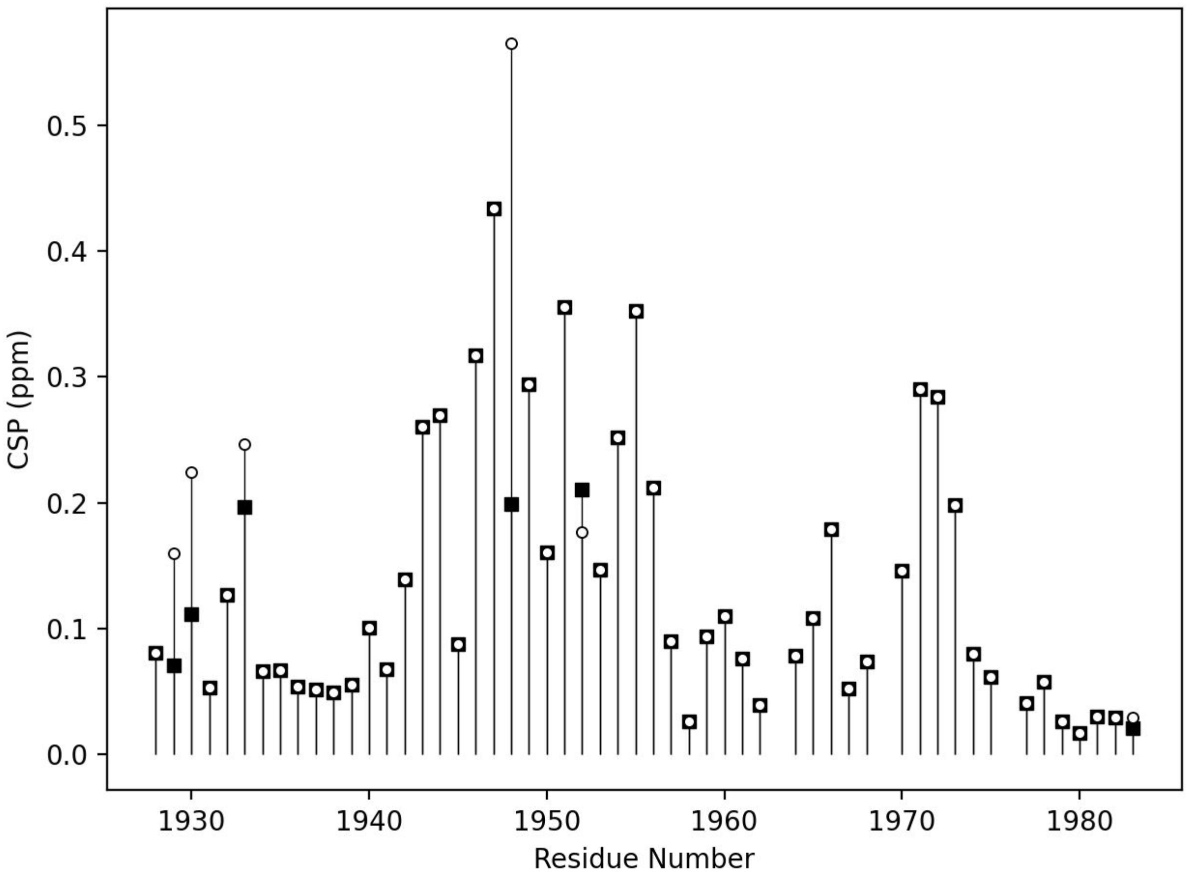

Supporting Figure S11 Comparison of experimental (black squares) and predicted (open circles) CSPs upon addition of ligand 10 to BAZ2B

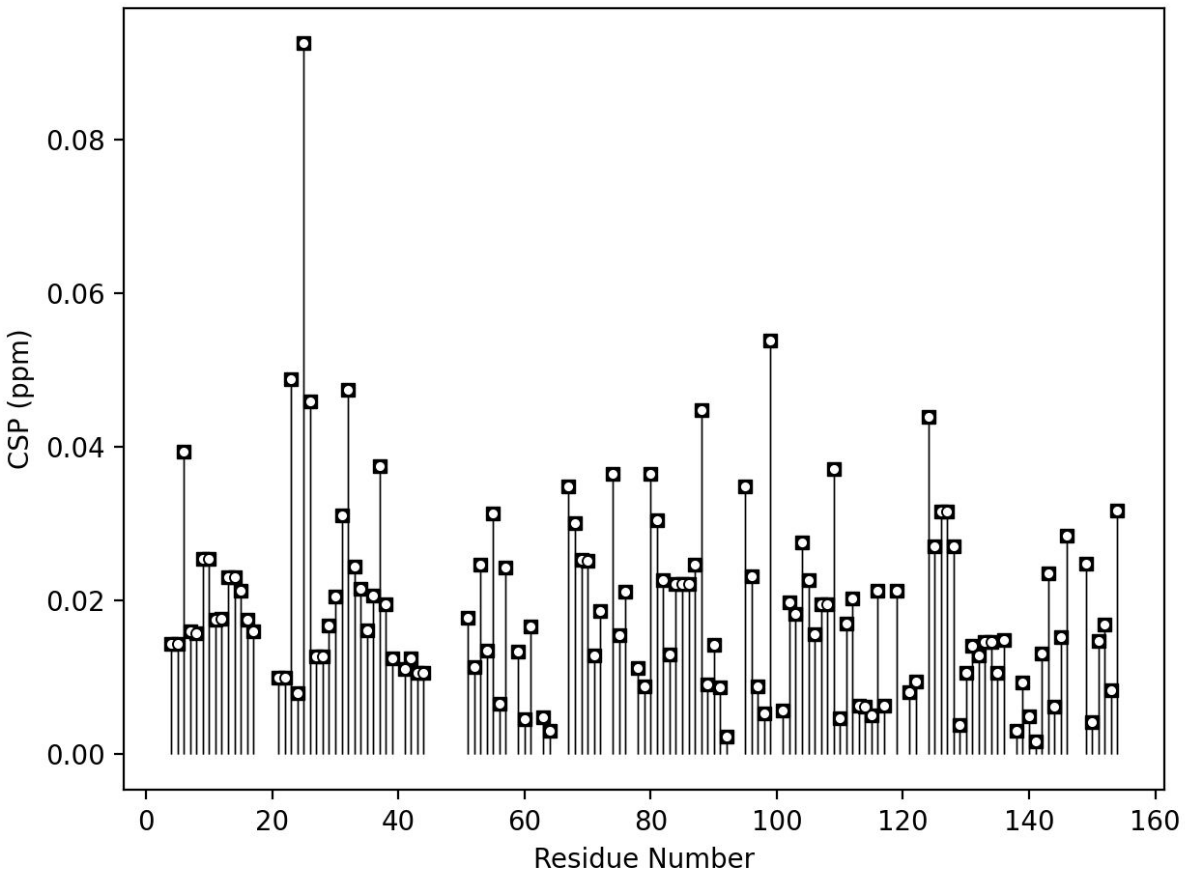

Supporting Figure S12 Comparison of experimental (black squares) and predicted (open circles) CSPs upon addition of ligand 11 to Ube2T

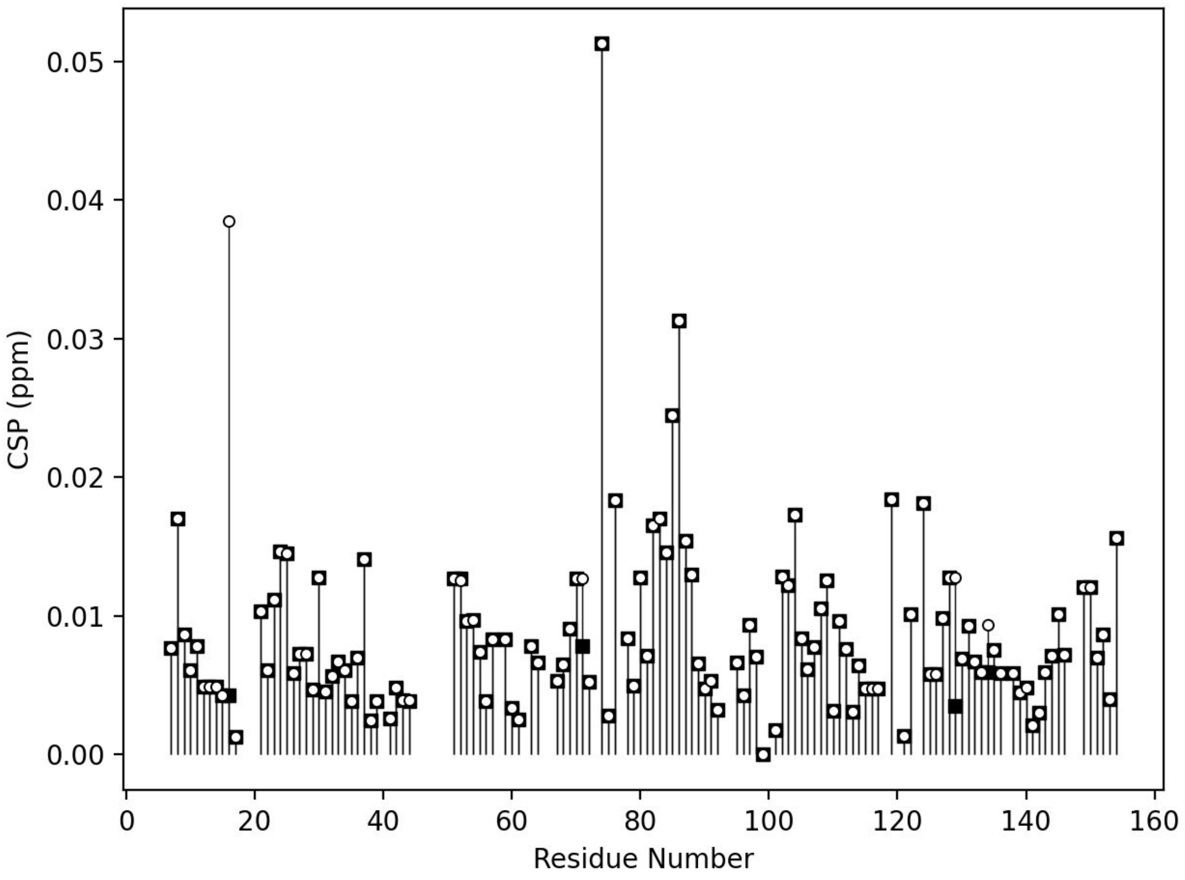

Supporting Figure S13 Comparison of experimental (black squares) and predicted (open circles) CSPs upon addition of ligand 12 to Ube2T

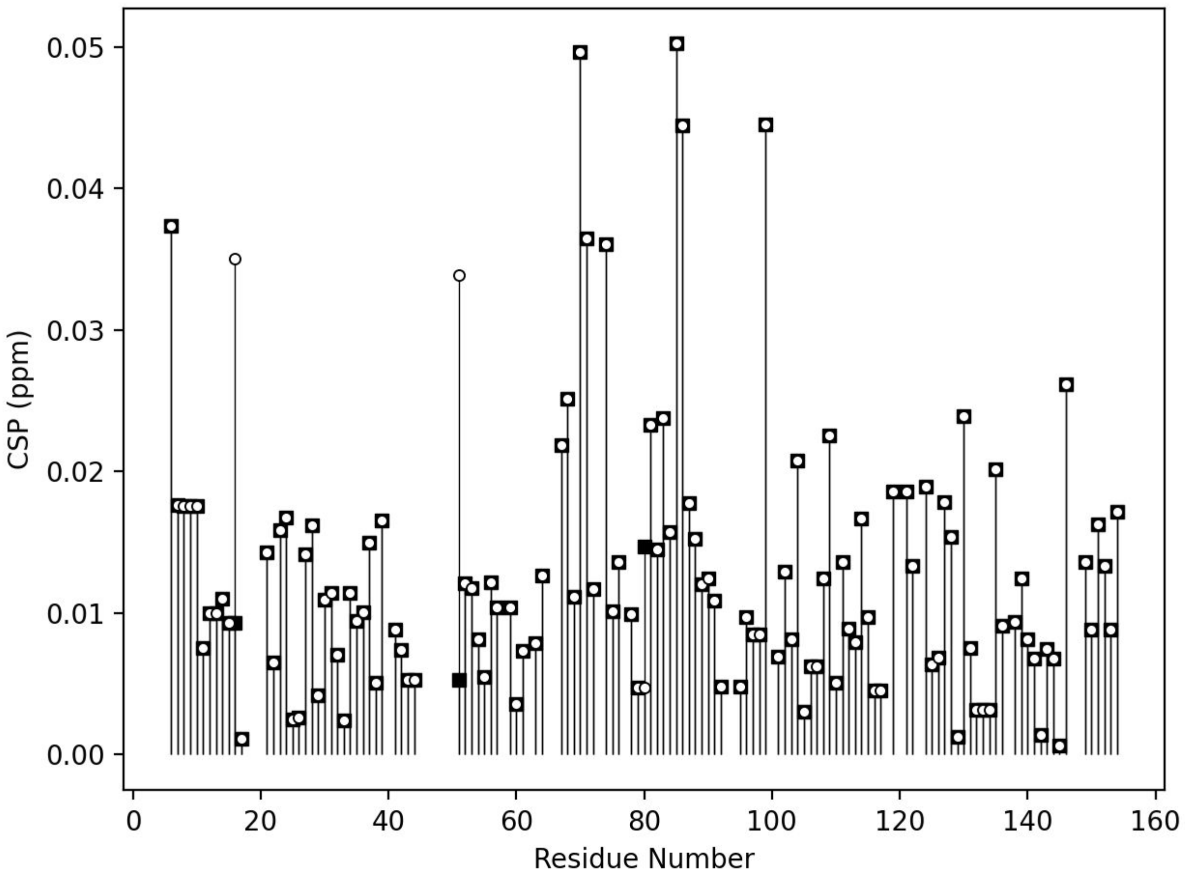

Supporting Figure S14 Comparison of experimental (black squares) and predicted (open circles) CSPs upon addition of ligand 13 to Ube2T

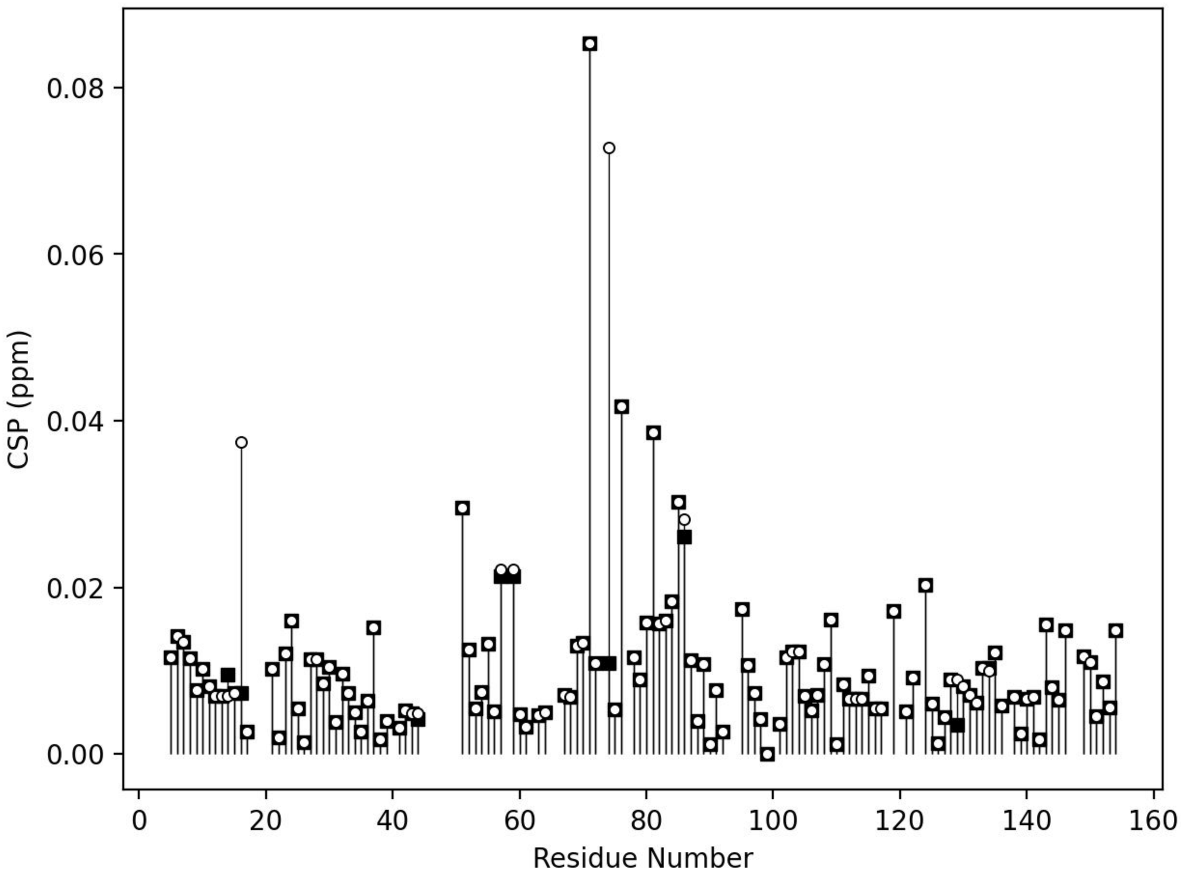

Supporting Figure S15 Comparison of experimental (black squares) and predicted (open circles) CSPs upon addition of ligand 14 to Ube2T.

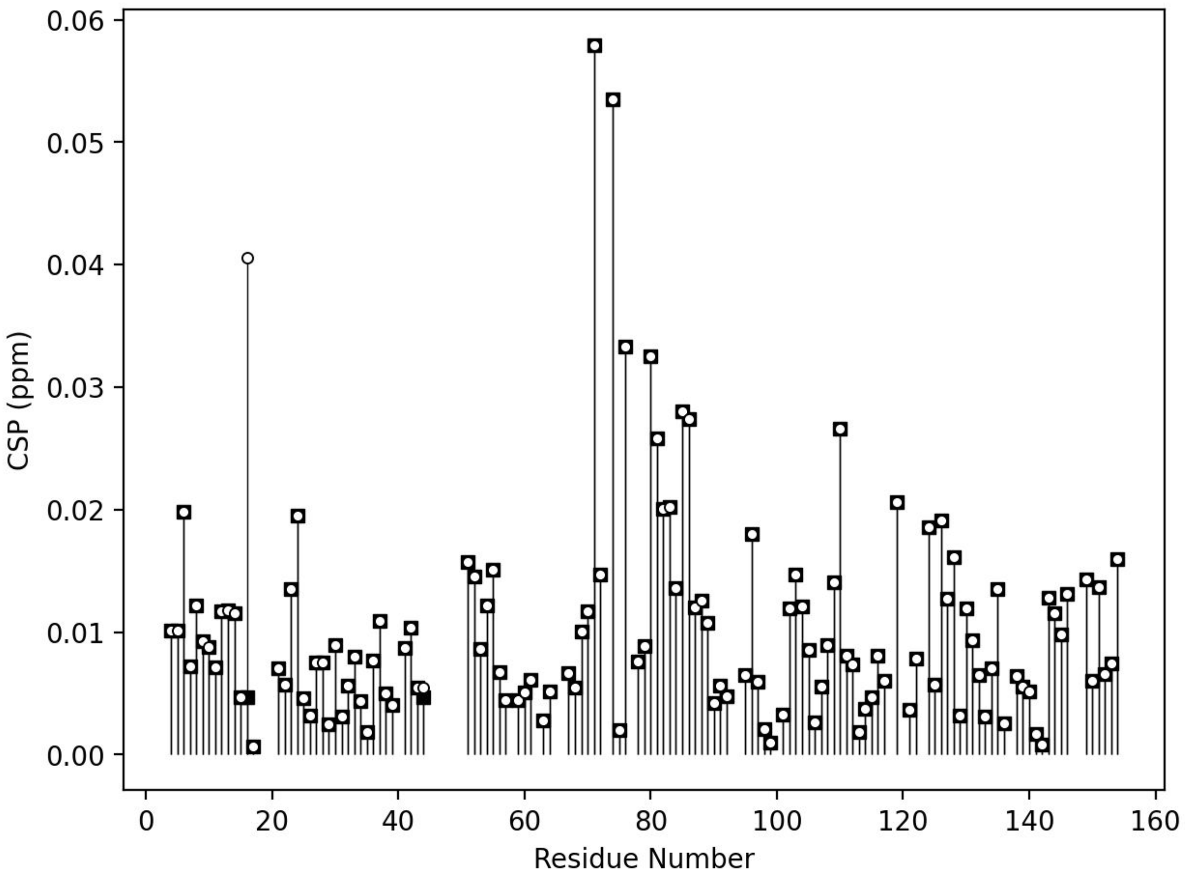

Supporting Figure S16 Comparison of experimental (black squares) and predicted (open circles) CSPs upon addition of ligand 15 to Ube2T

Supporting Tables.

Supporting Table S1. Format of input csv data to the Picasso web server. Top: for data with known assignment, use the amino acid one-letter code followed by the residue number without spaces; bottom: for data with unknown assignment, peaks must be numbered sequentially. The labels in the first row are read but not used.

```
Assignment, x, y
A115, 7.522, 120.917
A116, 8.095, 112.019
A133, 8.007, 124.602
A141, 8.829, 122.892
```

```
PeakNum, H, N
1, 7.524, 120.764
2, 8.127, 112.5
3, 7.969, 124.552
4, 8.902, 122.868
```

Supporting Table S2. Experimental ^1H and ^{15}N chemical shift values of carbonic anhydrase II in the presence of p-toluenesulfonamide in a 1:1 molar ratio with respect to the protein.

Residue	Number	^1H (ppm)	^{15}N (ppm)
Y	7	8.73	120.85
G	8	8.26	110.82
A	23	8.60	122.97
K	24	7.21	114.72
G	25	8.29	108.79
E	26	9.33	117.81
R	27	8.82	121.05
Q	28	7.67	115.45
S	29	8.16	118.52
V	31	6.31	106.91
D	32	8.28	118.15
I	33	8.72	128.44
D	34	7.41	128.45
T	35	10.28	122.38
H	36	8.63	119.81
T	37	7.59	108.14
A	38	7.50	127.64
K	39	8.13	122.51
Y	40	8.66	126.65
D	41	7.72	128.88
S	43	8.49	115.25
L	44	7.03	123.08
K	45	7.47	122.77
L	47	8.94	125.73
S	48	8.45	119.57
V	49	8.38	127.45
S	50	8.37	122.82
Y	51	8.97	125.50
D	52	8.87	121.49
Q	53	8.00	114.29
A	54	7.41	120.24
T	55	9.40	120.69
S	56	9.95	126.50
L	57	8.92	117.74
R	58	7.19	114.53
I	59	9.01	122.11
L	60	8.70	123.81
N	61	8.43	121.23
N	62	8.07	123.59

G	63	9.65	109.16
F	66	7.11	110.09
N	67	8.99	119.75
V	68	8.68	121.97
E	69	8.43	123.80
F	70	8.62	119.15
D	71	8.39	116.97
D	72	8.96	131.62
S	73	8.79	115.67
Q	74	7.67	117.65
K	76	7.99	124.28
A	77	8.20	123.23
V	78	8.39	115.87
L	79	9.04	123.67
K	80	8.35	121.05
G	81	8.97	106.41
G	82	7.07	108.68
L	84	7.75	120.31
G	86	7.85	109.44
T	87	8.48	116.70
Y	88	8.46	126.33
R	89	8.61	122.83
L	90	8.51	124.13
I	91	8.87	123.23
Q	92	7.35	115.38
F	93	8.63	113.42
H	94	8.14	113.56
W	97	9.64	119.48
G	98	7.99	107.60
S	99	8.42	111.83
L	100	7.36	119.04
D	101	8.88	121.65
G	102	7.66	102.76
Q	103	7.85	115.08
G	104	8.22	106.46
S	105	7.28	107.64
H	107	10.75	118.09
T	108	7.36	109.06
V	109	7.64	118.49
D	110	9.68	132.20
K	111	9.84	109.72
K	112	8.24	124.43
K	113	7.90	121.67

Y	114	8.15	121.29
A	115	7.60	120.85
A	116	8.15	112.28
E	117	9.50	121.72
L	118	9.84	130.51
H	119	8.85	126.31
L	120	9.04	123.90
V	121	9.16	126.25
H	122	8.58	124.45
W	123	9.07	119.20
N	124	9.06	119.86
T	125	8.15	117.17
K	126	7.90	122.75
Y	127	7.64	115.61
G	128	7.81	107.29
D	129	7.46	116.19
F	130	7.56	119.65
G	131	8.06	103.81
K	132	7.71	119.70
A	133	8.02	124.49
V	134	7.37	108.45
Q	135	6.57	113.79
Q	136	7.50	118.19
D	138	7.91	115.20
G	139	7.88	107.40
L	140	8.77	115.80
A	141	8.88	123.07
V	142	7.03	125.29
L	143	8.51	128.05
G	144	9.93	114.02
I	145	9.25	124.32
F	146	10.05	128.81
L	147	8.24	120.35
K	148	8.97	119.54
V	149	8.41	121.51
G	150	9.12	118.11
A	152	8.33	121.19
K	153	8.69	123.99
L	156	7.52	116.78
Q	157	7.78	122.28
K	158	8.72	115.58
V	159	7.02	113.21
V	160	7.29	114.43

D	161	8.37	117.65
V	162	7.14	113.99
L	163	7.06	121.65
D	164	8.16	116.27
S	165	7.99	113.54
I	166	7.39	116.31
K	167	7.33	117.37
T	168	6.58	98.71
K	169	7.45	120.57
G	170	8.82	116.79
K	171	7.77	120.18
S	172	8.29	113.64
A	173	9.00	123.33
D	174	8.58	122.09
F	175	7.97	125.27
T	176	8.07	114.90
N	177	9.69	115.55
F	178	8.24	118.92
D	179	7.96	128.11
R	181	8.37	118.43
G	182	7.29	102.64
L	183	7.24	117.40
L	184	6.61	113.10
E	186	8.18	116.08
S	187	7.59	111.33
L	188	8.62	125.47
D	189	7.39	121.94
Y	190	8.70	118.80
W	191	9.57	117.32
T	192	9.64	115.01
Y	193	8.12	125.81
G	195	9.37	110.85
S	196	8.72	121.03
T	199	7.09	107.88
L	202	9.19	120.76
L	203	5.85	111.02
E	204	8.72	124.62
C	205	7.56	114.44
V	206	7.15	119.15
T	207	8.38	124.99
W	208	8.19	129.83
I	209	8.75	127.35
V	210	9.66	128.41

L	211	9.01	126.79
K	212	7.36	122.17
E	213	8.88	121.78
I	215	8.93	113.30
S	216	8.06	116.69
V	217	8.53	115.92
S	218	8.41	116.07
S	219	9.28	117.41
E	220	8.35	118.27
Q	221	7.52	117.96
V	222	7.32	114.61
L	223	7.97	120.82
K	224	6.90	115.09
F	225	7.11	118.67
R	226	6.67	109.06
K	227	6.85	113.99
L	228	7.09	119.38
N	229	8.88	118.53
F	230	8.81	117.31
N	231	8.13	110.51
G	232	8.65	104.79
E	233	8.39	121.37
E	235	7.33	119.97
E	237	8.16	123.21
E	238	9.02	130.01
L	239	8.67	125.89
M	240	8.58	123.10
V	241	6.70	114.95
D	242	7.09	114.77
N	243	8.41	119.38
W	244	6.42	114.01
R	245	10.51	126.90
A	247	7.99	120.52
Q	248	8.75	122.02
L	250	8.65	125.69
K	251	8.85	114.56
R	253	7.30	118.53
Q	254	8.54	118.24
I	255	8.90	124.51
K	256	8.77	126.56
A	257	8.37	123.55
S	258	8.93	116.47
F	259	6.66	118.00

K	260	7.71	124.31

Supporting Table S3. Experimental ^{1}H and ^{15}N chemical shift values of carbonic anhydrase II in the presence of p-toluenesulfonic acid in a 100:1 molar ratio with respect to the protein.

Residue	Number	^{1}H (ppm)	^{15}N (ppm)
Y	7	8.67	120.42
G	8	8.25	110.96
A	23	8.60	122.92
K	24	7.16	114.79
G	25	8.25	108.84
E	26	9.33	117.74
R	27	8.80	120.68
Q	28	7.68	115.44
S	29	8.05	117.97
V	31	6.24	106.94
D	32	8.26	118.23
I	33	8.67	128.49
D	34	7.45	128.25
T	35	10.23	122.30
H	36	8.64	119.82
T	37	7.56	108.32
A	38	7.45	127.67
K	39	8.07	122.60
Y	40	8.65	126.63
D	41	7.69	128.88
S	43	8.44	115.26
L	44	6.97	123.10
K	45	7.47	122.98
L	47	8.92	125.96
S	48	8.42	119.79
V	49	8.35	127.55
S	50	8.33	122.82
Y	51	8.90	125.36
D	52	8.82	121.26
Q	53	7.98	114.41
A	54	7.36	120.16
T	55	9.36	120.64
S	56	9.93	126.41
L	57	8.86	117.65
R	58	7.13	114.66
I	59	8.99	122.12

L	60	8.65	123.68
N	61	8.41	121.33
N	62	8.00	123.33
G	63	9.66	108.88
F	66	7.17	110.12
N	67	8.97	119.59
V	68	8.59	121.82
E	69	8.38	123.93
F	70	8.55	118.91
D	71	8.37	116.98
D	72	8.93	131.70
S	73	8.78	115.79
Q	74	7.63	117.65
K	76	7.91	124.10
A	77	8.21	123.85
V	78	8.35	115.81
L	79	8.94	123.47
K	80	8.32	121.10
G	81	8.96	106.44
G	82	7.03	108.71
L	84	7.71	120.28
G	86	7.83	109.56
T	87	8.44	116.61
Y	88	8.43	126.14
R	89	8.54	122.70
L	90	8.49	124.05
I	91	8.84	123.12
Q	92	7.34	115.63
F	93	8.56	113.49
H	94	8.14	113.04
H	96	8.68	114.98
W	97	9.48	119.14
G	98	8.00	107.89
S	99	8.35	111.84
L	100	7.29	119.05
D	101	8.83	121.41
G	102	7.62	102.78
Q	103	7.81	115.02
G	104	8.20	106.28
S	105	7.27	107.75
E	106	8.26	119.18
H	107	10.66	117.87
T	108	7.31	109.31

V	109	7.58	118.55
D	110	9.65	132.25
K	111	9.80	109.73
K	112	8.19	124.52
K	113	7.90	121.84
Y	114	8.12	121.37
A	115	7.53	120.87
A	116	8.10	112.12
E	117	9.39	120.96
L	118	9.82	130.44
H	119	8.92	125.80
L	120	8.95	123.66
V	121	9.14	126.40
H	122	8.46	124.29
W	123	9.03	119.28
N	124	8.96	119.74
T	125	8.13	117.11
K	126	7.90	122.96
Y	127	7.54	115.49
G	128	7.78	107.43
D	129	7.43	116.26
F	130	7.49	119.72
G	131	8.01	103.90
K	132	7.67	119.67
A	133	8.00	124.59
V	134	7.35	108.05
Q	135	6.55	113.97
Q	136	7.51	118.25
D	138	7.84	114.86
G	139	7.89	107.62
L	140	8.77	115.92
A	141	8.85	122.90
V	142	7.16	124.42
L	143	8.42	128.01
G	144	9.86	114.05
I	145	9.10	124.31
F	146	10.06	128.94
L	147	8.18	120.29
K	148	8.92	119.31
V	149	8.39	121.46
G	150	9.07	118.11
A	152	8.32	121.23
K	153	8.65	123.87

L	156	7.49	116.76
Q	157	7.74	122.36
K	158	8.67	115.49
V	159	6.98	113.19
V	160	7.23	114.40
D	161	8.33	117.55
V	162	7.08	113.94
L	163	7.01	121.69
D	164	8.12	116.35
S	165	7.94	113.60
I	166	7.36	116.35
K	167	7.33	117.42
T	168	6.53	98.56
K	169	7.43	120.57
G	170	8.82	116.98
K	171	7.74	120.16
S	172	8.29	113.70
A	173	8.98	123.34
D	174	8.56	122.04
F	175	7.94	125.28
T	176	8.05	114.92
N	177	9.67	115.50
F	178	8.20	118.81
D	179	7.95	128.07
R	181	8.35	118.42
G	182	7.24	102.63
L	183	7.18	117.30
L	184	6.58	113.17
E	186	8.16	116.15
S	187	7.58	111.39
L	188	8.60	125.59
D	189	7.36	121.95
Y	190	8.68	119.31
W	191	9.51	117.20
T	192	9.62	115.11
Y	193	8.02	125.66
G	195	9.36	110.64
S	196	8.65	120.62
T	199	6.94	108.44
L	202	9.09	120.40
L	203	5.89	110.85
E	204	8.72	124.70
C	205	7.55	114.68

V	206	7.21	119.00
T	207	8.40	124.92
W	208	8.19	129.46
I	209	8.72	127.22
V	210	9.66	128.39
L	211	8.92	126.69
K	212	7.33	122.27
E	213	8.86	121.67
I	215	8.88	113.38
S	216	8.04	116.73
V	217	8.48	115.87
S	218	8.36	116.02
S	219	9.25	117.29
E	220	8.28	118.36
Q	221	7.48	117.94
V	222	7.32	114.81
L	223	7.97	120.86
K	224	6.86	115.06
F	225	7.06	118.75
R	226	6.64	109.08
K	227	6.80	114.13
L	228	7.06	119.30
N	229	8.86	118.44
F	230	8.81	117.19
N	231	8.10	110.61
G	232	8.62	104.79
E	233	8.34	121.33
E	235	7.30	120.03
E	237	8.19	123.36
E	238	8.99	130.11
L	239	8.68	125.93
M	240	8.57	123.05
V	241	6.67	114.87
D	242	7.10	114.74
N	243	8.36	119.37
W	244	6.42	114.13
R	245	10.36	127.01
A	247	7.94	120.37
Q	248	8.65	122.02
L	250	8.63	125.90
K	251	8.82	114.50
R	253	7.25	118.48
Q	254	8.53	118.25

I	255	8.87	124.38
K	256	8.73	126.56
A	257	8.33	123.61
S	258	8.91	116.46
F	259	6.61	117.99
K	260	7.70	124.36

Supporting Table S4. Experimental ^{1}H and ^{15}N chemical shift values of carbonic anhydrase II in the presence of thiocyanate in a 1250:1 molar ratio with respect to the protein.

Residue	Number	^{1}H (ppm)	^{15}N (ppm)
G	6	8.13	109.97
G	8	8.28	110.87
A	23	8.58	123.05
K	24	7.25	115.07
G	25	8.28	108.81
E	26	9.33	118.03
R	27	8.66	120.61
Q	28	7.71	115.59
S	29	8.15	118.48
V	31	6.29	106.75
D	32	8.29	118.17
I	33	8.70	128.41
D	34	7.45	128.22
T	35	10.27	122.22
T	37	7.62	109.03
A	38	7.48	127.31
K	39	8.17	122.87
Y	40	8.65	126.82
D	41	7.66	128.72
S	43	8.48	115.28
L	44	6.98	123.19
K	45	7.47	122.95
L	47	8.90	125.59
S	48	8.44	119.68
V	49	8.33	127.41
S	50	8.37	122.54
Y	51	8.96	124.50
D	52	8.80	120.70
Q	53	8.07	114.28
A	54	7.38	120.33
T	55	9.41	120.84

S	56	9.95	126.54
L	57	8.92	117.81
R	58	7.18	114.65
I	59	9.00	122.29
L	60	8.71	123.88
N	61	8.38	121.39
N	62	8.00	123.04
G	63	9.68	108.70
F	66	7.18	110.21
N	67	8.95	121.00
V	68	8.70	122.10
E	69	8.43	124.13
F	70	8.59	119.16
D	71	8.39	117.04
D	72	8.94	131.62
S	73	8.80	116.59
Q	74	7.67	117.79
K	76	7.91	123.84
A	77	8.24	123.64
V	78	8.34	116.05
L	79	8.96	123.47
K	80	8.37	120.91
G	81	8.95	106.52
G	82	7.05	108.78
L	84	7.77	120.28
G	86	7.83	109.39
T	87	8.43	116.13
Y	88	8.48	126.14
R	89	8.59	122.61
L	90	8.44	124.17
I	91	8.93	123.44
Q	92	7.31	115.30
F	93	8.53	113.32
H	94	8.16	113.30
F	95	9.11	116.86
H	96	8.68	115.58
W	97	9.52	119.10
G	98	8.06	107.86
S	99	8.39	112.12
L	100	7.42	119.32
D	101	8.85	121.54
G	102	7.58	103.39
Q	103	7.79	114.91

G	104	8.24	106.74
S	105	7.29	107.83
E	106	8.33	119.23
H	107	10.71	117.88
T	108	7.34	109.04
V	109	7.62	118.50
D	110	9.69	132.39
K	111	9.85	109.93
K	112	8.21	124.67
K	113	7.93	122.63
Y	114	8.13	121.05
A	115	7.60	121.21
A	116	8.13	112.06
E	117	9.40	121.31
L	118	9.85	130.44
H	119	8.88	125.71
L	120	8.99	124.06
V	121	9.07	126.50
H	122	8.48	124.05
W	123	9.04	119.05
N	124	9.04	119.67
T	125	8.14	117.19
K	126	7.91	122.87
Y	127	7.60	115.62
G	128	7.80	107.16
D	129	7.44	116.29
F	130	7.47	119.92
G	131	8.03	103.71
K	132	7.65	119.51
A	133	7.98	124.62
V	134	7.39	108.41
Q	135	6.55	113.79
Q	136	7.55	118.42
D	138	7.90	115.12
G	139	7.87	107.63
L	140	8.78	115.60
A	141	8.87	123.51
V	142	7.20	124.88
L	143	8.38	127.47
G	144	9.86	113.63
I	145	9.21	124.50
F	146	10.08	128.95
L	147	8.22	120.45

K	148	8.94	119.56
V	149	8.41	121.49
G	150	9.07	117.90
S	151	8.27	124.34
A	152	8.32	121.65
K	153	8.67	124.59
L	156	7.49	116.88
Q	157	7.82	122.36
K	158	8.62	115.72
V	159	7.03	113.50
V	160	7.26	114.47
D	161	8.37	117.58
V	162	7.12	113.84
L	163	7.07	121.63
D	164	8.17	115.95
S	165	7.98	113.63
I	166	7.40	116.46
K	167	7.38	117.51
T	168	6.56	98.67
K	169	7.44	120.45
G	170	8.98	117.17
K	171	7.78	120.22
S	172	8.29	113.56
A	173	8.96	123.04
D	174	8.57	122.02
F	175	7.97	125.33
T	176	8.06	114.85
N	177	9.66	115.58
F	178	8.23	119.04
D	179	7.94	128.33
R	181	8.38	118.73
G	182	7.29	102.93
L	183	7.23	117.46
L	184	6.60	113.00
E	186	8.12	116.46
S	187	7.62	111.39
L	188	8.59	125.59
D	189	7.34	121.79
Y	190	8.69	118.92
W	191	9.55	117.33
T	192	9.65	115.08
Y	193	8.10	125.75
G	195	9.38	110.86

S	196	8.59	119.88
T	199	6.98	108.50
L	202	9.17	120.52
L	203	5.96	110.91
E	204	8.71	124.52
C	205	7.60	114.51
V	206	7.18	119.31
T	207	8.41	125.04
W	208	8.17	129.71
I	209	8.79	127.44
V	210	9.65	128.37
L	211	9.04	126.99
K	212	7.32	122.17
E	213	8.86	121.82
I	215	8.89	113.22
S	216	8.05	116.41
V	217	8.47	115.86
S	218	8.35	115.97
S	219	9.25	117.34
E	220	8.31	118.18
Q	221	7.49	117.96
V	222	7.38	114.55
L	223	7.99	120.76
K	224	6.93	115.33
F	225	7.11	118.87
R	226	6.69	109.07
K	227	6.86	114.04
L	228	7.09	119.29
N	229	8.87	118.38
F	230	8.84	117.17
N	231	8.13	110.53
G	232	8.63	104.69
E	233	8.29	121.82
G	234	9.03	114.11
E	235	7.32	120.01
E	237	8.20	123.24
E	238	9.00	129.93
L	239	8.67	125.87
M	240	8.66	123.08
V	241	6.69	115.09
D	242	7.17	115.04
N	243	8.35	119.36
W	244	6.43	114.16

R	245	10.56	127.19
A	247	7.93	120.83
Q	248	8.71	122.01
L	250	8.63	125.72
K	251	8.80	114.70
R	253	7.28	118.47
Q	254	8.47	118.32
I	255	8.87	124.38
K	256	8.73	126.56
A	257	8.41	123.57
S	258	8.93	116.34
F	259	6.65	118.04
K	260	7.69	124.33
